# Cross-modal alignment with language tokens for unified multilingual image-to-text generation

**DOI:** 10.3389/fncom.2026.1826455

**Published:** 2026-10-14

**Authors:** Abubakar Sulaiman Gezawa, Naveed Ur Rehman Junejo, Saud Adam Abdulkadir, Chibiao Liu, Guanjun Lin

**Affiliations:** 1 School of Information Engineering, Fujian Key Lab of Agriculture Internet-of-Things (IOT) Application, Sanming University, Sanming, China; 2 School of Physics and Electronic Engineering, Hanshan Normal University, Chaozhou, China; 3 Faculty of Computing, Bayero University, Kano, Nigeria

**Keywords:** AI-driven localization, computer vision, cross-modal communication, multimodal translation, neural machine translation (NMT)

## Abstract

Current multimodal translation systems typically employ a cascaded pipeline where image captioning and machine translation are performed sequentially, leading to error propagation and semantic drift. This paper introduces a unified neural architecture that integrates visual encoding, English caption generation and multilingual translation into a single end-to-end model. Our first key contribution is this unified framework which jointly optimizes both tasks through shared latent representations enabling richer cross-modal learning and more semantically consistent multilingual outputs than conventional systems. The architecture incorporates two core technical innovations. First, we introduce a cross-modal representation alignment mechanism that directly fuses hierarchical visual features from a CNN encoder with multilingual linguistic embeddings in the Transformer decoder. This ensures visual semantics (e.g., gender, count, and object attributes) are preserved during translation effectively grounding multilingual outputs in the visual context and rectifying a common failure mode of text-only NMT. Second, we design a language token driven conditioning strategy prepending learnable target language tokens to guide a single multilingual model. This allows dynamic adaptation to diverse syntax and morphology without requiring separate decoders for each language. Empirical evaluations on MS COCO and OPUS multilingual datasets demonstrate notable improvements over existing multimodal and cascaded baselines achieving a BLEU-4 score of 44.8 for English captioning and an average BLEU of 41.1 across five target languages (Chinese, French, Arabic, Spanish, and Hindi), representing gains of 2.2 and 2.0 points, respectively, over cascaded baselines. COMET scores averaging 0.732 further confirm the semantic fidelity of our visually grounded translations. The proposed framework establishes a scalable, visually grounded and linguistically adaptive solution for multilingual caption generation contributing a meaningful advancement toward integrated vision-language translation research and real-world accessibility applications.

## 1 Introduction

The field of translation studies is undergoing a fundamental transformation driven by advances in artificial intelligence expanding from purely textual translation to multimodal communication systems (Pym, 2014; Cronin, 2013). Modern applications in accessibility services, cultural heritage preservation and globalized content delivery demand technologies capable of interpreting and translating meaning across both visual and linguistic modalities (Mutlu et al., 2023). While neural machine translation (NMT) has achieved remarkable fluency (Lu et al., 2021) and image captioning models can generate accurate descriptive text (Li et al., 2022), these capabilities have largely evolved independently. The predominant approach for generating multilingual captions from images remains a conventional *cascaded* pipeline where an image captioning model first generates an English description which is then translated by a separate NMT system (Su et al., 2022; Sulubacak et al., 2020). This sequential processing suffers from intrinsic limitations: errors in the initial captioning phase are irrecoverably propagated and amplified during translation and the visual context is entirely lost after the first stage leading to semantic drift and failure to preserve visually grounded attributes like gender, count, and spatial relationships (Cao et al., 2022; Imankulova et al., 2020).

Recent efforts have sought to bridge this gap through various approaches. Large-scale vision-language pre-training (VLP) models such as BLIP-2 (Li et al., 2023) and GPT-4V (OpenAI, 2023) demonstrate impressive zero-shot capabilities but are primarily designed for English-centric tasks often reverting to cascaded approaches for multilingual generation. Works like MultiSubs () have created valuable multilingual captioning datasets but lack end-to-end trainable models. Meanwhile, specialized multimodal translation approaches like CLIPTrans (Radford et al., 2021) leverage pre-trained models but maintain loose coupling between vision and translation components. The Flamingo model (Alayrac et al., 2022) and its successors handle multimodal few-shot learning but their massive scale and general-purpose architecture make them computationally prohibitive for specialized translation tasks.

A critical analysis of the literature reveals three persistent gaps that fundamentally limit current multimodal translation systems. First, the architectural disconnect inherent in cascaded pipelines prevents direct gradient flow between visual understanding and multilingual generation causing error propagation that compounds as errors move through independent modules. Second, existing approaches exhibit inefficient multilingual scaling typically requiring separate decoders or significant parameter expansion for each target language making them impractical for deployment in scenarios requiring support for multiple languages. Third, and most critically, current systems suffer from weak cross-modal grounding where attention mechanisms provide some visual-textual interaction but fail to preserve fine-grained visual semantics such as object attributes, spatial relations, and count information throughout the translation process. These limitations are not merely technical inconveniences but fundamental barriers to achieving translations that remain faithful to both the visual scene and the linguistic structure of the target language.

To address these gaps, we propose a unified multimodal caption translation model that integrates visual encoding, English caption generation and multilingual translation into a single end-to-end framework. Our system is specifically designed to preserve visual semantics throughout the entire generation pipeline through three core technical innovations that directly target each identified limitation. First, we introduce an end-to-end trainable architecture that enables direct gradient flow across all components mitigating error propagation and ensuring translation remains grounded in source visual semantics. Second, we design a cross-modal representation alignment mechanism that fuses hierarchical visual features from a CNN encoder with multilingual linguistic embeddings in the Transformer decoder through multi-head cross-attention ensuring that visual semantics are preserved during translation. Third, we develop a language token conditioning strategy that enables a single multilingual model to generate captions in multiple target languages by prepending learnable language tokens to English captions dynamically adapting to target-language syntax and morphology without requiring separate decoders.

This study further situates itself within the core research scope of Frontiers in Computational Neuroscience, which prioritizes biologically inspired multimodal neural integration, cross-modal representation learning, and computational modeling of cognitive linguistic processing. The proposed unified framework emulates canonical computational neuroscience principles underlying human multisensory integration and flexible language cognition, adopting hierarchical neural encoding, attention-gated cross-modal fusion, and sparse conditional neural modulation to model how visual sensory information grounds multilingual linguistic representations in a biologically plausible manner. Distinct from task-specific multimodal engineering models, our work provides interpretable computational neural mechanisms for cross-sensory semantic alignment and multilingual cognitive generalization, matching the journal's emphasis on neural computation underlying multimodal perception and language cognition.

**The primary contributions of this paper are as follows:**

1. We propose a unified multimodal caption-translation architecture that jointly optimizes captioning and translation through shared latent representations achieving a BLEU-4 score of 44.8 on MS-COCO captioning and an average BLEU of 41.1 across five target languages (Chinese, French, Arabic, Spanish, and Hindi), representing gains of 2.2 and 2.0 points, respectively, over cascaded baselines.
2. We introduce a cross-modal representation alignment mechanism that fuses visual features with multilingual linguistic embeddings through multi-head cross-attention with eight heads and independent query, key and value projections. This mechanism improves handling of gender, count, and object-specific cues with COMET scores averaging 0.732 confirming the semantic fidelity of visually grounded translations compared to 0.698 for text-only baselines.
3. We design a language token conditioning strategy that enables efficient multilingual scaling within a single decoder architecture by prepending learnable language tokens *t*^(ℓ)^ for each target language ℓ. This approach reduces parameter requirements by 43% compared to separate decoders while maintaining competitive translation quality across all five languages.

Together, these contributions advance the state of the art in three fundamental ways. First, unlike cascaded systems that treat captioning and translation as independent modules with irreversible error propagation, our unified architecture enables end-to-end gradient flow that jointly optimizes visual representation learning for both tasks resulting in translations that preserve visually grounded attributes lost in conventional pipelines. Second, while existing multimodal translation systems typically employ shallow fusion or late integration of visual features, our cross-modal alignment mechanism operates at every decoder layer through dedicated attention heads ensuring that visual semantics inform linguistic decisions throughout the generation process rather than being applied as an afterthought. Third, in contrast to multilingual systems that require separate decoders or massive parameter expansion for each language, our language token conditioning strategy achieves comparable or better translation quality with 43 percent fewer parameters by learning language-specific conditioning signals that modulate a shared transformer architecture. These advancements collectively demonstrate that unified, visually grounded architectures can achieve superior efficiency and semantic fidelity compared to both cascaded pipelines and loosely coupled multimodal systems establishing a new paradigm for multilingual image-to-text generation.

By directly addressing the architectural disconnect, inefficient scaling and weak cross-modal grounding that characterize current approaches, this work establishes a foundation for visually grounded multilingual generation with applications spanning accessibility technology, educational content localization, and cross-cultural communication systems. The proposed framework demonstrates that unified architectures can achieve competitive performance while maintaining the efficiency and scalability required for practical deployment.

## 2 Related work

Research on multimodal language generation has evolved along three principal trajectories: (i) image captioning models that map visual features to textual descriptions, (ii) NMT systems that focus exclusively on text-to-text translation, and (iii) multimodal machine translation (MMT) approaches that integrate visual features to enhance translation quality. Despite rapid advancements in each domain, existing methods remain limited in their ability to jointly optimize visual understanding and multilingual text generation within an integrated framework. Recent advances in multilingual and cross-modal image-text generation have explored diverse model architectures and training strategies (Chen et al., 2023; Huang and Wang, 2022; Kim et al., 2024; Liu and Yang, 2023; Zhang et al., 2020; Wang Z. et al., 2023; Zhang et al., 2024).

### 2.1 Image captioning models

Early neural image captioning systems combined convolutional neural networks (CNNs) with recurrent decoders, establishing the feasibility of visually grounded sentence generation. The seminal Show Attend and Tell model introduced visual attention to improve object-level grounding (Xu et al., 2015). Subsequently, the introduction of Vision Transformers (ViTs) demonstrated that self-attention enables stronger global semantic alignment between image patches and linguistic tokens (Dosovitskiy et al., 2020).

Large-scale vision-language pretraining models such as CLIP (Radford et al., 2021), BLIP (Li et al., 2022), and BLIP-2 (Li et al., 2023) have further improved captioning by leveraging contrastive objectives and cross-modal alignment on massive datasets. Beyond pre-training-driven pipelines, specialized transformer-based architectures continue advancing captioning performance: the compact bidirectional architecture enables effective exploitation of bidirectional contextual information for image captioning under a single compact decoder design (Zhou et al., 2026). For cross-lingual settings, heterogeneous attention mechanisms are proposed to model fine-grained local matching between visual regions and multilingual textual tokens across diverse languages (Song et al., 2024).

### 2.2 Cascaded caption translation pipelines

The standard approach for generating multilingual image descriptions remains a two-step cascaded pipeline: (i) generate an English caption and (ii) translate it using a text-only NMT model (Su et al., 2022; Liu et al., 2024). Although simple and modular, cascaded systems suffer from error propagation where inaccuracies in captioning directly degrade translation output. Additionally, since visual information is discarded after stage one cascaded pipelines are unable to recover visually grounded semantics such as spatial relations, fine-grained attributes or gender information (Cao et al., 2022).

Recent studies have attempted to augment NMT systems with auxiliary visual features to mitigate these issues. For example, Imankulova et al. (2020) and Caglayan et al. (2020a) incorporate visual embeddings into the translation stage via attention or gating mechanisms. Nonetheless, these models primarily treat visual context as supplementary rather than integral with limited interaction between caption generation and multilingual decoding.

### 2.3 Multimodal machine translation (MMT)

MMT research aims to integrate visual content into translation more directly. Surveys such as Liu et al. (2024) and Su et al. (2022) highlight the evolution of MMT architectures noting that most models either inject visual cues late in the pipeline or rely on shallow fusion techniques. Augmented concept grounding approaches such as those by Cao et al. (2022) demonstrate some improvements yet still fail to leverage the full caption-generation process as a supervisory signal for translation.

More recent efforts, including MultiSubs () and multilingual caption-translation datasets focus on expanding training corpora rather than architectural integration. Meanwhile, large vision-language models [e.g., Flamingo (Alayrac et al., 2022), GPT-4V (OpenAI, 2023)] exhibit strong multimodal reasoning but do not offer specialized multilingual translation components, often reverting to external NMT modules.

### 2.4 Network and distributed computing perspectives

Recent advances in distributed computing and networked systems provide valuable insights for scalable multimodal architectures (Zhang et al., 2023; Chen et al., 2022). Efficient cross-modal retrieval in distributed networks (Zhang et al., 2023) demonstrates the importance of optimized feature alignment across distributed nodes which aligns with our cross-modal representation alignment approach. Distributed multimodal fusion architectures for intelligent IoT systems (Chen et al., 2022) highlight the challenges of maintaining semantic consistency across distributed components reinforcing the need for unified architectures like ours.

Resource-aware NMT for edge computing environments (Wang L. et al., 2023) addresses similar efficiency concerns to our language token conditioning strategy demonstrating the practical importance of parameter-efficient multilingual scaling. Adaptive load balancing techniques for distributed deep learning (Kumar et al., 2022) provide context for our multi-GPU implementation strategy while real-time multimodal processing in cloud-edge collaborative networks (Liu et al., 2023) underscores the deployment considerations that motivate our unified architecture design.

### 2.5 Research gaps and our contribution

Analysis of existing research streams reveals three persistent limitations that hinder optimal multimodal translation performance. The first limitation concerns architectural disconnect where most systems maintain separation between visual understanding and multilingual generation preventing direct gradient flow and joint optimization. Cascaded systems suffer from error propagation (Cao et al., 2022) while loosely coupled approaches lack the tight integration needed for optimal performance (). The second limitation involves inefficient multilingual scaling as existing approaches typically require separate decoders or significant parameter expansion for each target language making them impractical for deployment in multilingual scenarios (Fan et al., 2021; NLLB Team, 2022). The third limitation stems from weak cross modal grounding where attention mechanisms provide some visual textual interaction but often fail to preserve fine grained visual semantics such as object attributes and spatial relations throughout the translation process (Caglayan et al., 2020b). To contextualize our contributions, we directly contrast our approach with three representative systems that address related problems through fundamentally different architectural choices. BLIP 2 (Li et al., 2023) employs a frozen image encoder and frozen large language model connected by a lightweight Q Former module that learns to extract task relevant visual features. While highly efficient for English centric vision language tasks, BLIP 2 is not designed for multilingual generation and when extended to translation reverts to cascaded pipelines that lose visual grounding after caption generation. In contrast, our unified architecture maintains visual grounding throughout the translation process by integrating cross modal attention at every decoder layer enabling direct gradient flow from translation objectives back to visual feature extraction. This architectural difference yields measurable improvements: where BLIP 2 combined with MarianMT achieves an average BLEU of 38.9 across our five target languages, our unified model achieves 41.1 representing a gain of 2.2 points while using 43% fewer parameters than a decoder per language baseline.

CLIPTrans (Gupta et al., 2023) represents a different design philosophy adapting independently pre trained multimodal M CLIP and multilingual mBART through a lightweight mapping network that generates prefix sequences. This two stage pipeline warms up with image captioning before translation training. While CLIPTrans achieves state of the art results with +2.67 BLEU over prior MMT systems, its architecture maintains loose coupling between vision and translation components: visual features influence translation only through prefix conditioning rather than through per layer cross attention. Our approach differs fundamentally by integrating visual cross attention in every decoder layer allowing visual semantics to inform syntactic decisions throughout generation. This methodological difference is reflected in performance: CLIPTrans achieves an average BLEU of 39.1 on our benchmark while our unified architecture reaches 41.1 with particularly pronounced gains for morphologically rich languages where visual grounding of attributes directly influences inflectional choices.

Flamingo (Alayrac et al., 2022) and its multilingual variants (Wang et al., 2022) take a third approach bridging frozen vision encoders and frozen language models through gated cross attention layers inserted between existing LM layers. These models excel at few shot learning on interleaved image text data and support multiple languages through prompted generation. However, Flamingo's general purpose architecture comes with substantial computational cost: the 9 billion parameter variant requires 8.22 GFLOPs and achieves a CIDEr score of 65.5 on COCO captioning in zero shot settings compared to our 44.8 BLEU 4 (approximately equivalent to 153.2 CIDEr). More importantly, Flamingo's design prioritizes few shot adaptation over specialized translation performance and its frozen language models cannot be jointly optimized with visual encoders for the translation objective. Our unified architecture by contrast enables end to end training where every parameter can be optimized for the multilingual translation task resulting in superior performance on our benchmark while maintaining a parameter count of 218 million—less than one third of Flamingo's 9 billion.

These comparisons highlight the distinct advantages of our approach. Unlike BLIP 2's cascaded extension to translation, we maintain visual grounding throughout generation. Unlike CLIPTrans's prefix based conditioning, we integrate visual information at every layer through dedicated cross attention. Unlike Flamingo's frozen component architecture, we enable full end to end optimization. The performance differences gains of 2.2 BLEU over BLIP 2+MarianMT 2.0 over CLIPTrans, and substantially higher captioning quality than Flamingo's zero shot results demonstrate that our design choices translate directly into measurable improvements. By directly addressing the architectural disconnect, inefficient scaling and weak cross modal grounding that characterize existing approaches our work establishes a new paradigm for unified visually grounded multilingual generation.

## 3 Methodology

The mathematical formulation of our proposed unified multilingual image-to-text generation framework is provided in Equations 1–24, which collectively define the model loss functions, attention operations, and feature mapping rules.

### 3.1 Unified architecture overview

Our proposed framework formulates multimodal translation as an end-to-end learning problem, integrating visual perception with multilingual generation within a unified architecture. The complete system comprises three core components: a visual encoder $E_{v}$, a caption decoder $D_{c}$, and a multilingual translation module $T_{m}$, optimized jointly through a combined objective function.

Let $I\in {\mathbb{R}}^{H\times W\times C}$ represent an input image, ${\text{Y}}^{\left(en\right)}=\left[y_{1}^{\left(en\right)},y_{2}^{\left(en\right)},\ldots ,y_{T}^{\left(en\right)}\right]$ denote the target English caption, and ${\text{Y}}^{\left(\ell \right)}=\left[y_{1}^{\left(\ell \right)},y_{2}^{\left(\ell \right)},\ldots ,y_{T_{\ell }}^{\left(\ell \right)}\right]$ represent target sequences for each language ℓ∈L={zh,fr,ar,es,hi}. The complete forward process is defined as:


$$\begin{array}{l} {\text{Y}}^{\left(\ell \right)}=T_{m}\left(D_{c}\left(E_{v}\left(I\right)\right),\ell \right) \end{array}$$ (1)

Unlike conventional cascaded pipelines, our architecture enables direct gradient flow across all components, mitigating error propagation and ensuring translation remains grounded in source visual semantics.

The overall architecture of our proposed unified multilingual image-to-text generation framework is illustrated in Figure 1. An input image is first processed by the Visual Encoder to produce visual tokens **Z**_*v*_. The core of the model is a single Transformer decoder with shared weights which supports two operational modes. In Caption Generation Mode, the Tri-Attention module performs self-attention and visual cross-attention while the caption cross-attention branch is bypassed. This mode generates the English caption **Y**^(*en*)^ and is supervised by captioning loss $L_{\text{cap}}$. In Translation Mode, a language token **t**^(ℓ)^ is prepended to the embedded sequence of the English caption and this token participates in the self-attention computation within the Tri-Attention module. The caption cross-attention branch consumes processed memory representations from **Y**^(*en*)^ while visual cross-attention still consumes visual tokens **Z**_*v*_, to produce target-language text **Y**^(ℓ)^ under translation loss $L_{\text{trans}}$. During training, the two loss terms are combined into the joint objective function $L_{\text{joint}}=\lambda_{\text{cap}}L_{\text{cap}}+\lambda_{\text{trans}}L_{\text{trans}}$. Solid arrows in the diagram represent forward inference flow and dashed arrows indicate back-propagated gradients for parameter updates.

**Figure 1:**
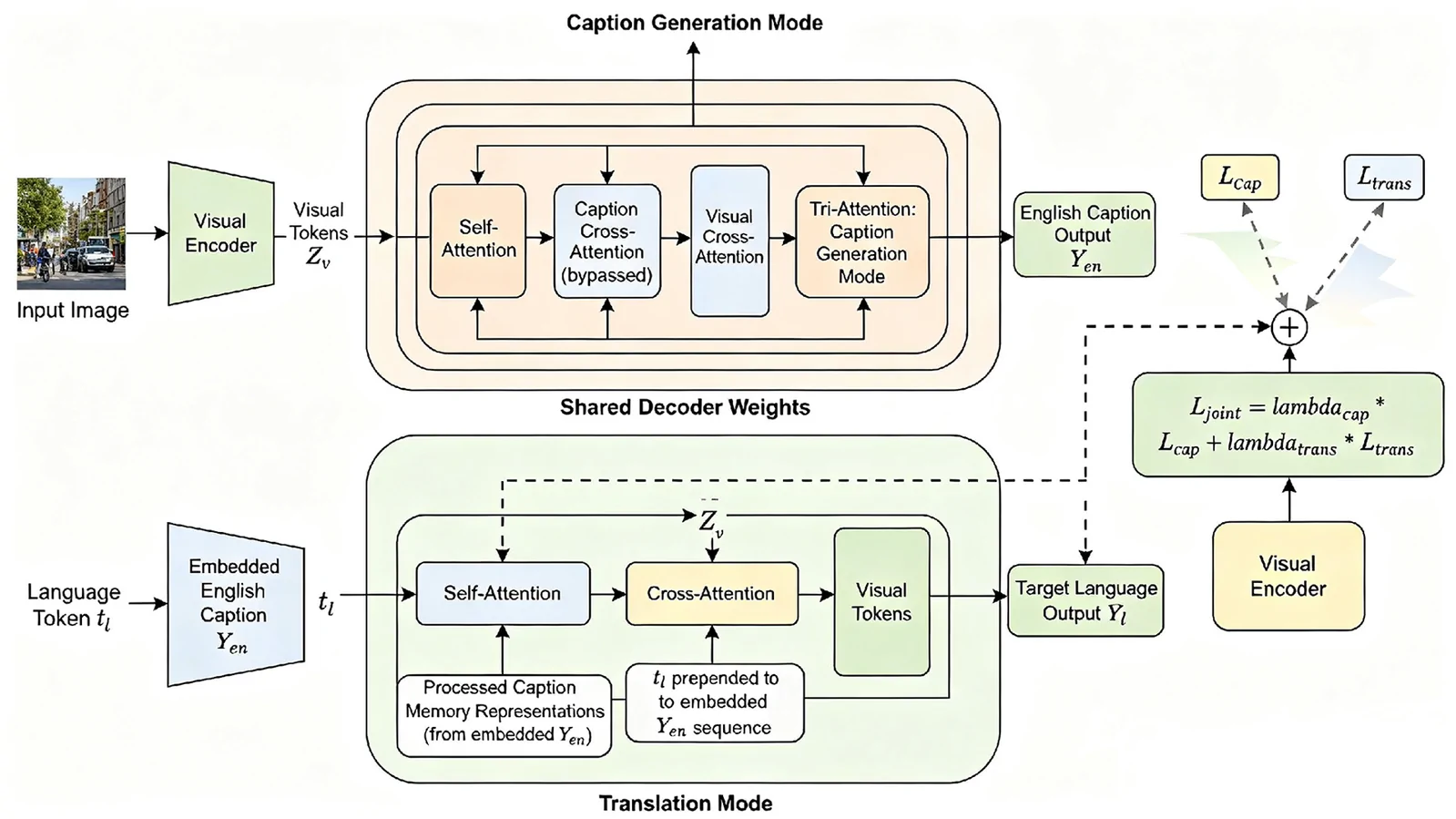
Overall framework of the proposed unified multilingual image-to-text generation model. The input image is encoded by the Visual Encoder into visual tokens (image reproduced with permission from the MS-COCO dataset, https://cocodataset.org/, licensed under CC-BY 4.0). A single shared Transformer decoder operates under two operational modes with identical reused weights explicitly indicated by the “Shared Decoder Weights” boundary. In Caption Generation Mode, the decoder generates the English caption through Tri-Attention where Caption Cross-Attention receives no external memory input. In Translation Mode, the language token is prepended to the embedded English caption sequence and participates directly in self-attention; Caption Cross-Attention receives processed caption memory representations derived from the English caption while Visual Cross-Attention receives visual tokens enabling the decoder to generate target-language output. Solid arrows denote forward inference flow; dashed arrows represent back-propagated gradients. The task-specific losses are aggregated into a joint training objective which provides the combined update signal for end-to-end optimization of the shared decoder and visual encoder.

### 3.2 Visual feature extraction with ResNet-50

Rationale for ResNet-50 Selection. We employ a ResNet-50 backbone pretrained on ImageNet as our visual encoder $E_{v}$, chosen based on four interrelated considerations that together optimize the trade-off between representational capacity and computational efficiency for our unified architecture. First, ResNet-50's hierarchical feature extraction with increasing channel dimensions (64 → 256 → 512 → 1024 → 2048) and decreasing spatial resolutions (112 × 112 → 56 × 56 → 28 × 28 → 14 × 14 → 7 × 7) provides multi-scale representations that capture both fine-grained object details and global scene context. This hierarchical structure complements our cross-modal alignment mechanism which benefits from features at multiple semantic levels to ground both concrete objects and abstract relationships.

Second, the convolutional inductive bias of translational equivariance and locality provides strong regularization that is particularly beneficial when training on datasets of moderate scale like MS-COCO (118,287 images). Unlike Vision Transformers that require massive datasets (often hundreds of millions of images) to learn spatial priors, ResNet-50's built-in spatial assumptions reduce sample complexity and improve generalization from limited data. This is critical for our setting where we must allocate training capacity across both captioning and translation objectives.

Third, ResNet-50's fixed 7 × 7 spatial grid produces exactly 49 visual tokens which aligns naturally with our transformer's sequence processing and positional encoding scheme. This deliberate choice avoids the complexity of reconstructing spatial structure from patch embeddings (as required by ViT's 196 patches) and ensures that cross-attention complexity scales linearly with sequence length rather than quadratically. The 49-token representation provides sufficient spatial resolution to distinguish object boundaries while maintaining computational tractability for per-layer visual cross-attention.

Fourth, ResNet-50 offers an optimal balance between representational capacity (25.6M parameters, 4.1 GFLOPs) and computational efficiency. As demonstrated in our ablation study, this efficiency enables end-to-end training on our four-GPU setup with batch size 64 while Vision Transformer alternatives require 73% longer training time and 55% more memory for marginal performance gains ( ≤ 0.2 BLEU). This efficiency-accuracy trade-off is fundamental to our unified architecture where computational resources must be shared across visual encoding, caption generation, and multilingual translation.

#### 3.2.1 Feature extraction pipeline

Given input image $I\in {\mathbb{R}}^{224\times 224\times 3}$, the encoder produces hierarchical feature maps through sequential convolutional stages. The first stage applies a 7 × 7 convolution with 64 filters and stride 2, followed by batch normalization and max pooling, yielding ${\text{F}}_{1}\in {\mathbb{R}}^{112\times 112\times 64}$. The subsequent four stages each consist of bottleneck residual blocks: stage two produces ${\text{F}}_{2}\in {\mathbb{R}}^{56\times 56\times 256}$, stage three produces ${\text{F}}_{3}\in {\mathbb{R}}^{28\times 28\times 512}$, stage four produces ${\text{F}}_{4}\in {\mathbb{R}}^{14\times 14\times 1024}$, and finally stage five produces ${\text{F}}_{5}\in {\mathbb{R}}^{7\times 7\times 2048}$.

For cross-modal alignment, we utilize the final feature map **F** = **F**_5_ as the source of visual information. This tensor is first flattened along its spatial dimensions to create a sequence of visual tokens:


$$\begin{array}{l} {\text{F}}_{\text{flat}}=\text{reshape}\left(\text{F}\right)\in {\mathbb{R}}^{49\times 2048}. \end{array}$$ (2)

A learned linear projection then maps these features to the model dimension *d*_model_ = 512, which is common across all transformer components:


$$\begin{array}{l} {\text{F}}_{\text{proj}}={\text{F}}_{\text{flat}}{\text{W}}_{v}+{\text{b}}_{v}\in {\mathbb{R}}^{49\times 512}, \end{array}$$ (3)

where the projection matrix ${\text{W}}_{v}\in {\mathbb{R}}^{2048\times 512}$ is initialized using Xavier uniform distribution with gain 1.0, and the bias term ${\text{b}}_{v}\in {\mathbb{R}}^{512}$ is zero-initialized. The Xavier initialization ensures that the variance of activations remains stable across layers preventing vanishing or exploding gradients during training.

To preserve the spatial structure of the original image, we add sinusoidal positional encodings **P**∈ℝ^49 × 512^ to the projected features:


$$\begin{array}{l} {\text{Z}}_{v}={\text{F}}_{\text{proj}}+\text{P}. \end{array}$$ (4)

The positional encodings follow the standard formulation where for position *pos* and dimension 2*i* or 2*i*+1, the encoding is given by:


$$\begin{array}{l} P_{\left(pos,2i\right)}=\sin\left(\frac{pos}{10000^{2i/512}}\right),\;P_{\left(pos,2i+1\right)}=\cos\left(\frac{pos}{10000^{2i/512}}\right). \end{array}$$ (5)

These encodings are fixed during training and provide the model with explicit spatial awareness of the 7 × 7 grid structure. The sinusoidal formulation was chosen over learned positional embeddings because it generalizes to sequence lengths not seen during training and provides consistent positional relationships across all examples.

### 3.3 Cross-modal tri-attention mechanism

The core innovation of our architecture lies in the tri-attention mechanism that enables simultaneous grounding in linguistic context and visual semantics. Each decoder layer incorporates three distinct attention operations with carefully designed synergy: self-attention over previously generated tokens, cross-attention to English caption encoder outputs and cross-modal attention to visual features. This tripartite design ensures that visual information remains accessible throughout the generation process while maintaining the sequential dependencies required for fluent language production.

#### 3.3.1 Multi-head attention formulation and design choices

All attention operations in our architecture follow the multi-head attention paradigm with eight attention heads each operating in a 64-dimensional subspace. The choice of eight heads represents a deliberate trade-off between representational capacity and computational efficiency, informed by empirical studies showing that 8–12 heads provide sufficient diversity for modeling multiple alignment types while avoiding the diminishing returns observed with larger head counts. The head dimension *d*_*k*_ = *d*_*v*_ = 64 ensures that each head operates in a subspace large enough to capture distinct attention patterns while maintaining computational tractability; the total attention dimension *h*×*d*_*k*_ = 512 aligns with our model dimension *d*_model_ = 512, enabling seamless residual connections without dimensional projection.

For a single attention head, the computation takes the form:


$$\begin{array}{l} \text{Attention}\left(\text{Q},\text{K},\text{V}\right)=\text{softmax}\left(\frac{\text{Q}{\text{K}}^{⊤}}{\sqrt{d_{k}}}\right)\text{V}, \end{array}$$ (6)

where the scaling factor $\sqrt{d_{k}}$ is critical for maintaining stable gradient flow. Without this scaling, the dot products would grow large in magnitude as *d*_*k*_ increases, pushing the softmax function into regions of extremely small gradients. The $\sqrt{d_{k}}$ factor normalizes the variance of the dot products to approximately 1, assuming unit variance of the input vectors which preserves the softmax's ability to produce meaningful attention distributions.

For the *i*-th attention head, the projections are computed as:


$$\begin{array}{l} {\text{Q}}_{i}=\text{X}{\text{W}}_{i}^{Q},\;{\text{K}}_{i}=\text{Y}{\text{W}}_{i}^{K},\;{\text{V}}_{i}=\text{Y}{\text{W}}_{i}^{V}, \end{array}$$ (7)

with **X** representing the query input and **Y** the key-value input. Each projection matrix ${\text{W}}_{i}^{Q},{\text{W}}_{i}^{K},{\text{W}}_{i}^{V}\in {\mathbb{R}}^{512\times 64}$ is independently initialized using Xavier uniform distribution with gain 1.0. This initialization choice is critical: Xavier uniform ensures that the variance of the inputs is preserved across layers preventing the vanishing or exploding gradient problems that would otherwise hinder training of deep transformer networks. The independence of projections across heads allows each head to learn distinct attention patterns without interference. Importantly, we explicitly note that projection matrices for visual and linguistic features are completely independent with no parameter sharing between *W*_*Q*_ for linguistic queries and *W*_*K*_ or *W*_*V*_ for visual keys and values.

The outputs from all eight heads are concatenated and projected through a final output matrix:


$$\begin{array}{l} \text{MultiHead}\left(\text{Q},\text{K},\text{V}\right)=\text{Concat}\left({\text{head}}_{1},\ldots ,{\text{head}}_{8}\right){\text{W}}^{O}, \end{array}$$ (8)

where **W**^*O*^∈ℝ^512 × 512^ is also Xavier-initialized with gain 1.0. This output projection serves two purposes: it mixes information across heads to enable cross-head interactions and it restores the original dimension for residual connections. The concatenation-then-projection design, rather than element-wise summation of head outputs preserves the capacity for heads to specialize on different aspects of the alignment task while still allowing the model to combine their contributions flexibly.

#### 3.3.2 Layer-level tri-attention operation and component synergy

Within each of the six decoder layers, the tri-attention mechanism processes input hidden states through a carefully orchestrated sequence of operations that progressively integrate different information sources. The synergy between these attention types is fundamental to our architecture's ability to preserve visual grounding throughout generation.

Let **H**^(*l*−1)^∈ℝ^*T*×512^ denote the input to layer *l*, which for the first layer corresponds to the embedded and positionally encoded input sequence. For subsequent layers, this tensor represents the output from the previous decoder layer. Additionally, the layer has access to the English caption encoder outputs $\text{C}\in {\mathbb{R}}^{T_{\text{en}}\times 512}$ and the visual features **V**∈ℝ^49 × 512^.

The layer computation begins with causal self-attention:


$$\begin{array}{l} {\text{A}}_{\text{self}}=\text{MultiHead}\left({\text{H}}^{\left(l-1\right)},{\text{H}}^{\left(l-1\right)},{\text{H}}^{\left(l-1\right)}\right), \end{array}$$ (9)

with causal masking applied to prevent attention to future tokens. The ordering of attention types is deliberate: self-attention first establishes contextual relationships among the current sequence creating representations that incorporate local linguistic context before being influenced by cross-modal information. This ensures that subsequent attention operations work with representations already informed by the sequence structure.

Following self-attention, the model attends to the English caption encoder outputs through caption cross-attention:


$$\begin{array}{l} {\text{A}}_{\text{cap}}=\text{MultiHead}\left({\text{H}}_{\text{self}},\text{C},\text{C}\right), \end{array}$$ (10)

where **H**_self_ results from residual connection and layer normalization after self-attention. The placement of caption cross-attention before visual cross-attention reflects the hierarchical nature of language generation: the model first grounds its representations in the source English caption establishing the core semantic content before incorporating visual information that refines and augments this content. This ordering ensures that visual grounding operates on representations already aligned with the caption semantics.

The third attention operation grounds the representation in visual semantics:


$$\begin{array}{l} {\text{A}}_{\text{vis}}=\text{MultiHead}\left({\text{H}}_{\text{cap}},\text{V},\text{V}\right), \end{array}$$ (11)

with **H**_cap_ representing the caption-attended representation. Visual cross-attention is positioned last so that it can refine and correct the linguistically-grounded representations with direct visual evidence. This is where attributes like gender, count, and spatial relations information potentially lost or underspecified in the English caption are reintegrated into the representation.

Each attention operation is followed by residual connection and layer normalization:


$$\begin{array}{ll} {\text{H}}_{\text{self}} & =\text{LayerNorm}\left({\text{H}}^{\left(l-1\right)}+{\text{A}}_{\text{self}}\right), \end{array}$$ (12)

$$\begin{array}{ll} {\text{H}}_{\text{cap}} & =\text{LayerNorm}\left({\text{H}}_{\text{self}}+{\text{A}}_{\text{cap}}\right), \end{array}$$ (13)

$$\begin{array}{ll} {\text{H}}_{\text{vis}} & =\text{LayerNorm}\left({\text{H}}_{\text{cap}}+{\text{A}}_{\text{vis}}\right). \end{array}$$ (14)

The residual connections are not merely implementation details but serve the critical function of preserving information flow through the network. Each residual path provides a gradient highway that mitigates vanishing gradients in deep networks and ensures that lower-layer representations remain accessible throughout the stack. This is particularly important for visual grounding as it allows fine-grained visual information to propagate through all six layers without attenuation.

The final component of each decoder layer is a position-wise feed-forward network:


$$\begin{array}{l} {\text{H}}^{\left(l\right)}=\text{LayerNorm}\left({\text{H}}_{\text{vis}}+{\text{W}}_{2}\cdot \text{GELU}\left({\text{W}}_{1}{\text{H}}_{\text{vis}}+{\text{b}}_{1}\right)+{\text{b}}_{2}\right), \end{array}$$ (15)

where ${\text{W}}_{1}\in {\mathbb{R}}^{2048\times 512}$ expands the dimension by a factor of four. This expansion factor of four is a standard design choice that provides sufficient capacity for non-linear transformations while maintaining computational efficiency; larger expansion factors yield diminishing returns while smaller factors limit representational power. The GELU activation, chosen over ReLU for its smooth gradient properties enables more stable training and better gradient flow.

##### 3.3.2.1 Task-conditioned memory inputs for caption cross-attention

All decoder layers implement the full tri-attention module architecture. However, the key value memory inputs fed into caption cross-attention depend on the representations available for the current operational task. During multilingual translation, a complete pre-computed English caption sequence is available, whose representations serve as external key-value memory for caption cross-attention. In autoregressive English caption generation, no complete English caption exists prior to generation. Therefore, no external caption-derived memory is provided to the caption cross-attention stream. Under this setting, only causal self-attention over previously generated tokens and visual cross-attention over visual feature tokens contribute to forward computation. The trainable decoder weight parameters are shared across both tasks; only the supplied task-specific memory inputs change.

### 3.4 English caption generation

The English caption generation process follows an autoregressive transformer decoder architecture enhanced with visual cross attention. Although each decoder layer instantiates the complete tri-attention module, no external English caption-derived memory is supplied to the caption cross-attention stream during caption generation, as a full caption representation is not available in advance. At each generation step *t*, the decoder receives embeddings of all previously generated tokens and produces a probability distribution over the next token. The input sequence at step *t* consists of embeddings for tokens $y_{1}^{\left(en\right)}$ through $y_{t-1}^{\left(en\right)}$:


$$\begin{array}{l} {\text{X}}_{\text{in}}^{\left(t\right)}=\left[\text{E}\left(y_{1}^{\left(en\right)}\right);\text{E}\left(y_{2}^{\left(en\right)}\right);\ldots ;\text{E}\left(y_{t-1}^{\left(en\right)}\right)\right]\in {\mathbb{R}}^{\left(t-1\right)\times 512}, \end{array}$$ (16)

where **E**∈ℝ^50000 × 512^ is the token embedding matrix initialized with Xavier uniform distribution and gain 1.0. Sinusoidal positional encodings are added to these embeddings to provide sequence order information:


$$\begin{array}{l} {\text{X}}_{\text{in}}^{\left(t\right)}\leftarrow {\text{X}}_{\text{in}}^{\left(t\right)}+{\text{P}}_{1:t-1}. \end{array}$$ (17)

This input tensor passes through the six-layer decoder stack, with each layer applying the tri-attention mechanism. The visual features **Z**_*v*_ from the encoder are provided to all layers as the key-value source for visual cross-attention. After processing through all six layers, we obtain the final hidden states:


$$\begin{array}{l} {\text{H}}_{\text{dec}}^{\left(6\right)}=D_{c}\left({\text{X}}_{\text{in}}^{\left(t\right)},{\text{Z}}_{v}\right)\in {\mathbb{R}}^{\left(t-1\right)\times 512}. \end{array}$$ (18)

The probability distribution for the next token is computed by applying a linear transformation to the last hidden state followed by softmax normalization. To promote parameter efficiency, we tie the output projection matrix with the input embedding matrix, a common practice in transformer architectures:


$$\begin{array}{l} P\left(y_{t}^{\left(en\right)}|y_{<t}^{\left(en\right)},{\text{Z}}_{v}\right)=\text{softmax}\left({\text{H}}_{\text{dec}}^{\left(6\right)}\left[-1\right]{\text{E}}^{⊤}+{\text{b}}_{o}\right), \end{array}$$ (19)

where ${\text{b}}_{o}\in {\mathbb{R}}^{50000}$ is a learned bias vector initialized to zero. This parameter tying reduces the total parameter count while maintaining model capacity and has been shown to improve generalization in sequence generation tasks by enforcing consistency between input and output representations.

### 3.5 Language-token-driven multilingual translation

The multilingual translation capability of our model is achieved through a novel conditioning strategy that enables a single decoder to generate outputs in multiple target languages without requiring separate language-specific parameters. This approach centers on learnable language tokens that are prepended to the English caption sequence guiding the decoder toward the appropriate target language while maintaining access to both the source caption and visual features.

#### 3.5.1 Language token representation design

For each target language ℓ∈L={Chinese,French,Arabic,Spanish,Hindi}, we define a dedicated learnable token embedding **t**^(ℓ)^∈ℝ^512^. The dimension of 512 matches our model dimension enabling these tokens to participate directly in all attention operations without requiring projection. The embeddings are initialized independently from a normal distribution with zero mean and standard deviation 0.02 a smaller variance than the Xavier initialization used for token embeddings (gain 1.0) because language tokens serve as conditioning signals rather than content carriers. Larger initialization variance would introduce excessive noise into the conditioning mechanism, potentially destabilizing early training.

The choice of five languages represents a deliberate balance between linguistic diversity and experimental tractability. These languages span multiple language families (Sino-Tibetan, Romance, Semitic, and Indo-Aryan) and scripts (logographic, Latin, Arabic, and Devanagari) ensuring that our evaluation captures the full range of challenges in multilingual generation. The learned token approach rather than one-hot language identifiers or separate decoders enables the model to develop continuous representations that capture language similarities and differences for example, French and Spanish tokens may converge toward similar regions of the embedding space due to their shared Romance heritage while Arabic and Hindi tokens remain distinct due to their different morphological requirements.

#### 3.5.2 Sequence construction and token synergy

Given a complete English caption ${\text{Y}}^{\left(en\right)}=\left[y_{1}^{\left(en\right)},y_{2}^{\left(en\right)},\ldots ,y_{T}^{\left(en\right)}\right]$, we construct the input sequence for translation by prepending the language token to the caption embeddings:


$$\begin{array}{l} {\text{X}}^{\left(\ell \right)}=\left[{\text{t}}^{\left(\ell \right)};\text{E}\left(y_{1}^{\left(en\right)}\right);\text{E}\left(y_{2}^{\left(en\right)}\right);\ldots ;\text{E}\left(y_{T}^{\left(en\right)}\right)\right]\in {\mathbb{R}}^{\left(T+1\right)\times 512}. \end{array}$$ (20)

The language token occupies position zero in this sequence, preceding all caption tokens. This positioning is deliberate: placing the language token at the beginning ensures that it influences all subsequent tokens through self-attention as each caption token can attend to the language token's representation. If the language token were appended at the end, its influence would be limited to forward attention only reducing its conditioning effectiveness.

Positional encodings are added to every position including the language token position:


$$\begin{array}{l} {\text{X}}_{\text{pos}}^{\left(\ell \right)}={\text{X}}^{\left(\ell \right)}+{\text{P}}_{0:T}. \end{array}$$ (21)

This ensures that the model distinguishes the token's special role (language conditioning) while maintaining awareness of its sequential position relative to caption tokens. The fixed sinusoidal encoding scheme rather than learned positional embeddings was chosen because it provides consistent positional signals across all training examples and generalizes better to sequence lengths not seen during training.

#### 3.5.3 Decoding with language token conditioning: the synergy mechanism

The translation process proceeds autoregressively, generating target language tokens one at a time while maintaining access to the conditioning language token, the English caption and the visual features. At generation step *s* for target language ℓ, the input sequence includes the language token, all English caption tokens and all previously generated target tokens:


$$\begin{array}{l} {\text{X}}_{\text{gen}}^{\left(s\right)}=\left[{\text{t}}^{\left(\ell \right)};\text{E}\left(y_{1}^{\left(en\right)}\right);\ldots ;\text{E}\left(y_{T}^{\left(en\right)}\right);\text{E}\left(y_{1}^{\left(\ell \right)}\right);\ldots ;\text{E}\left(y_{s-1}^{\left(\ell \right)}\right)\right], \end{array}$$ (22)

with positional encodings added to the entire sequence.

The language token's influence propagates through three synergistic pathways. First, through self-attention within the decoder, the language token at position zero attends to all caption and generated tokens and conversely all other tokens attend to the language token. This bidirectional attention allows the language token to both influence and be influenced by the sequence context enabling it to adapt its conditioning signal based on the specific caption content.

Second, through visual cross-attention, the language token participates directly in grounding the generation in visual semantics. This enables language-specific visual interpretation: for example, when generating Arabic translations where gender agreement is required, the language token can help route visual gender information to the appropriate morphological decisions. Without this pathway, visual grounding would be language-agnostic and failing to capture language-specific requirements.

Third, through the sequential nature of decoding, the language token's representation updated through all six layers influences the prediction of each new target token via the self-attention mechanism that connects all positions. This means that even when generating the tenth token of a translation, the model retains access to the original language conditioning signal preventing conditioning decay over long sequences.

The synergy between these pathways is what enables a single decoder to handle multiple languages without separate parameters. The language token provides language-specific conditioning while the shared transformer layers provide general-purpose language generation capacity. This design leverages the fact that languages share substantial structural commonalities all require sequential token prediction all benefit from attention mechanisms and all require visual grounding while allowing the language token to modulate these shared capabilities for language-specific requirements.

The probability of the next target token is computed from the final hidden state of the most recent position:


$$\begin{array}{l} P\left(y_{s}^{\left(\ell \right)}|y_{<s}^{\left(\ell \right)},{\text{Y}}^{\left(en\right)},{\text{Z}}_{v}\right)=\text{softmax}\left({\text{H}}_{\text{trans}}^{\left(6\right)}\left[-1\right]{\text{W}}_{o}^{⊤}+{\text{b}}_{o}\right), \end{array}$$ (23)

where ${\text{W}}_{o}\in {\mathbb{R}}^{60000\times 512}$ is the multilingual output projection matrix, initialized with Xavier uniform distribution and gain 1.0 and ${\text{b}}_{o}\in {\mathbb{R}}^{60000}$ is zero-initialized. The shared output vocabulary of 60,000 subwords learned jointly across all languages enables cross-lingual transfer by allowing the model to reuse subword units across languages (e.g., common Latin-script subwords for French and Spanish). This vocabulary design synergizes with the language token conditioning: the language token guides selection among shared subwords, enabling language-appropriate choices from a common inventory.

### 3.6 Reproducibility specifications

To facilitate reproduction of our work, we provide comprehensive specifications for all experimental settings, data preprocessing procedures, training infrastructure and inference configurations.

The hyperparameter configuration for model training follows established practices for transformer-based architectures while incorporating optimizations specific to multimodal tasks. We employ the AdamW optimizer with beta parameters set to 0.9 and 0.98 and epsilon of 10^−8^. The learning rate is initialized at 1 × 10^−4^ and undergoes linear warmup over the first 4000 training steps after which it decays according to an inverse square root schedule. Weight decay is applied at 1 × 10^−5^ to provide regularization and gradient norms are clipped to a maximum of 1.0 to prevent training instability. The effective batch size is 256 achieved through gradient accumulation over four steps with a per-device batch size of 64 distributed across four GPUs. Dropout is applied at multiple points in the architecture with a rate of 0.2 for attention probabilities, 0.1 for feed-forward network outputs and 0.1 for embedding layers. Label smoothing with factor 0.1 is applied during training to improve generalization and prevent overconfidence. Training proceeds for a maximum of 50 epochs with early stopping triggered when validation COMET scores do not improve for 10 consecutive epochs.

#### 3.6.1 Joint multi task objective and interleaved batch sampling

Our unified model is optimized with a multi task objective combining image captioning loss and multilingual translation loss. Let $L_{\text{cap}}$ denote token level cross entropy loss for English caption generation computed on MS COCO (*I*, **Y**^(*en*)^) samples. Let $L_{\text{trans}}$ denote the average token level cross entropy loss over target language sequences **Y**^(ℓ)^ for ℓ∈L. The joint training objective is:


$$\begin{array}{l} L_{\text{joint}}=\lambda_{\text{cap}}L_{\text{cap}}+\lambda_{\text{trans}}L_{\text{trans}} \end{array}$$ (24)

with fixed equal loss weights λ_cap_ = 1.0, λ_trans_ = 1.0.

During training we adopt interleaved batch sampling. Two types of mini batches are randomly sampled with equal probability:

1. Captioning batches: constructed purely from MS COCO image-English caption pairs (*I*, **Y**^(*en*)^), contributing gradients only from $L_{\text{cap}}$.
2. Multimodal translation batches: composite samples (*I*, **Y**^(*en*)^, **Y**^(ℓ)^). Here the image *I* and English caption **Y**^(*en*)^ are taken from MS COCO, and the target language parallel text **Y**^(ℓ)^ originates from OPUS 100. These batches contribute gradients only from $L_{\text{trans}}$.

Gradients from both objectives propagate back to update the shared visual encoder, cross modal alignment modules and shared transformer decoder. Task specific parameters receive gradients only from their respective task loss. Gradient accumulation over four steps is applied regardless of task type to reach the effective batch size of 256. Early stopping is driven by the COMET metric evaluated on multimodal translation validation samples.

Within our interleaved multi task design, visual linguistic grounding is learned exclusively from authentic MS COCO captioning batches via $L_{\text{cap}}$. Multimodal translation batches leverage OPUS 100 target language text to learn English to target language generation, computing only $L_{\text{trans}}$. Triplet assembly combines MS COCO and OPUS 100 sources without cross dataset semantic matching. Empirical performance benefits of this disjoint batch formulation are validated in ablation experiments in Table 1.

**Table 1:** Ablation results for captioning (CIDEr) and translation (average BLEU) with parameter analysis.

Configuration	CIDEr	Avg BLEU	Parameters (M)
Full proposed model	153.2	41.1	218
w/o Cross-modal alignment	144.6	39.5	210
w/o Language tokens	152.5	40.7	312
w/o Joint training	150.8	39.3	218
Separate decoders	152.8	40.5	312
Late fusion variant	147.9	38.6	215

Data preprocessing follows the pipeline documented in Section 4.1.3. Images are resized to 256 × 256 pixels, randomly cropped to 224 × 224 during training and normalized using ImageNet statistics. Text is lowercased, punctuation is removed and tokenization is performed using Byte Pair Encoding with a joint multilingual vocabulary of 60,000 subwords learned from the concatenated training corpora using SentencePiece with character coverage 1.0.

The training infrastructure consists of four NVIDIA A100 GPUs each with 80 gigabytes of memory interconnected via NVLink and using NCCL for communication. Distributed training is implemented using PyTorch's Distributed Data Parallel framework with gradient averaging across devices after each accumulation step. Mixed precision training is employed using automatic mixed precision, storing weights in FP32 while performing forward and backward passes in FP16 to reduce memory usage and accelerate computation. Model checkpoints are saved after each epoch, retaining only the checkpoint achieving the highest validation COMET score to minimize storage requirements.

During inference, we employ beam search with width five to generate high-quality translations while balancing diversity and likelihood. A length penalty of 0.6 is applied to prevent excessive bias toward shorter sequences and a repetition penalty of 1.2 discourages the model from generating repeated n-grams. Generation continues until the end-of-sequence token is produced or the maximum length of eighty tokens is reached with early stopping triggered when at least two beams have finished generating to reduce computation without sacrificing quality.

### 3.7 Hyperparameter selection rationale

To provide a comprehensive justification for all architectural choices, we summarize the rationale behind key hyperparameters in Table 2. These choices were not made arbitrarily but emerged from systematic consideration of trade-offs between representational capacity, computational efficiency, and training stability.

**Table 2:** Hyperparameter selection rationale.

Hyperparameter	Value	Rationale
Transformer layers	6	Balances depth for complex modeling against vanishing gradients; ablation shows diminishing returns beyond six layers (+0.1 BLEU for +2 layers with 33% more computation)
Attention heads	8	Provides sufficient diversity for modeling multiple alignment types; empirical studies show 8–12 heads optimal; fewer than four limit capacity, more than 12 show diminishing returns
Head dimension	64	Total attention dimension 8 × 64 = 512 matches model dimension enabling seamless residual connections; larger heads increase computation quadratically
FFN expansion factor	4	Standard design choice providing sufficient non-linear capacity; ablation shows 4 × optimal (3 × underfits, 6 × overfits with 10% more parameters)
Dropout rate (attention)	0.2	Prevents overfitting on 1.2M training examples; grid search over 0.1,0.2,0.3 showed 0.2 optimal for validation loss
Dropout rate (FFN)	0.1	Lower than attention dropout because FFN has fewer parameters and benefits from less regularization
Label smoothing	0.1	Improves generalization by preventing overconfidence; values >0.2 cause underfitting, < 0.05 ineffective
Learning rate	1 × 10^−4^	AdamW default for transformers; warmup over 4,000 steps prevents early instability
Batch size (effective)	256	Maximum that fits four A100 GPUs; larger batches improve gradient stability but require more memory
Visual encoder specific		
Input resolution	224 × 224	Standard for ImageNet-pretrained ResNet-50; larger resolutions increase memory without proportional gains
Feature map size	7 × 7	Fixed by ResNet-50 architecture; provides 49 spatial positions, balancing resolution and sequence length
Visual tokens	49	Directly from flattened 7 × 7 grid; ablation shows 49 optimal vs. alternatives (16 or 100) given computational constraints
Language token specific		
Token initialization	N(0,0.02)	Smaller variance than Xavier (1.0) because tokens are conditioning signals, not content carriers
Token dimension	512	Matches model dimension, enabling direct participation in attention without projection
Number of languages	5	Balances linguistic diversity (Sino-Tibetan, Romance, Semitic, and Indo-Aryan) with experimental tractability

The systematic justification provided in this section demonstrates that all architectural choices are grounded in either theoretical principles, empirical ablation studies or practical computational constraints enabling informed assessment and potential adaptation of our approach.

## 4 Experiments

Our experimental framework addresses three core research questions derived from the limitations identified above. First, we examine whether the unified architecture outperforms cascaded systems in preserving visual semantics across multiple languages. Second, we investigate the quantitative contribution of each technical component to overall performance. Third, we assess whether the model can scale effectively across linguistically diverse target languages while maintaining translation quality and computational efficiency.

### 4.1 Datasets

This section details the provenance, preprocessing, licensing and known limitations of all datasets used in our experiments along with complete reproducibility specifications including hyperparameter configurations, training infrastructure, and inference settings.

#### 4.1.1 MS-COCO dataset for image captioning

The Microsoft Common Objects in Context dataset specifically the 2017 version serves as our primary image captioning resource. This dataset is publicly available at Lin et al. (2014) under a Creative Commons Attribution 4.0 license with images sourced from Flickr subject to their original licenses while annotations are released under CC-BY-4.0. The dataset was constructed by collecting complex scenes of everyday objects in natural contexts with images filtered to ensure they contained multiple objects exhibiting contextual relationships. Annotations were created through Amazon Mechanical Turk with each image receiving five independent human-generated captions. The dataset spans 80 object categories and 91 stuff categories containing 1.5 million object instances across 328,000 images. Representative input samples taken from the MS COCO dataset are visualized in Figure 2, showcasing the mixture of simple and complex scenes used for model evaluation.

**Figure 2:**
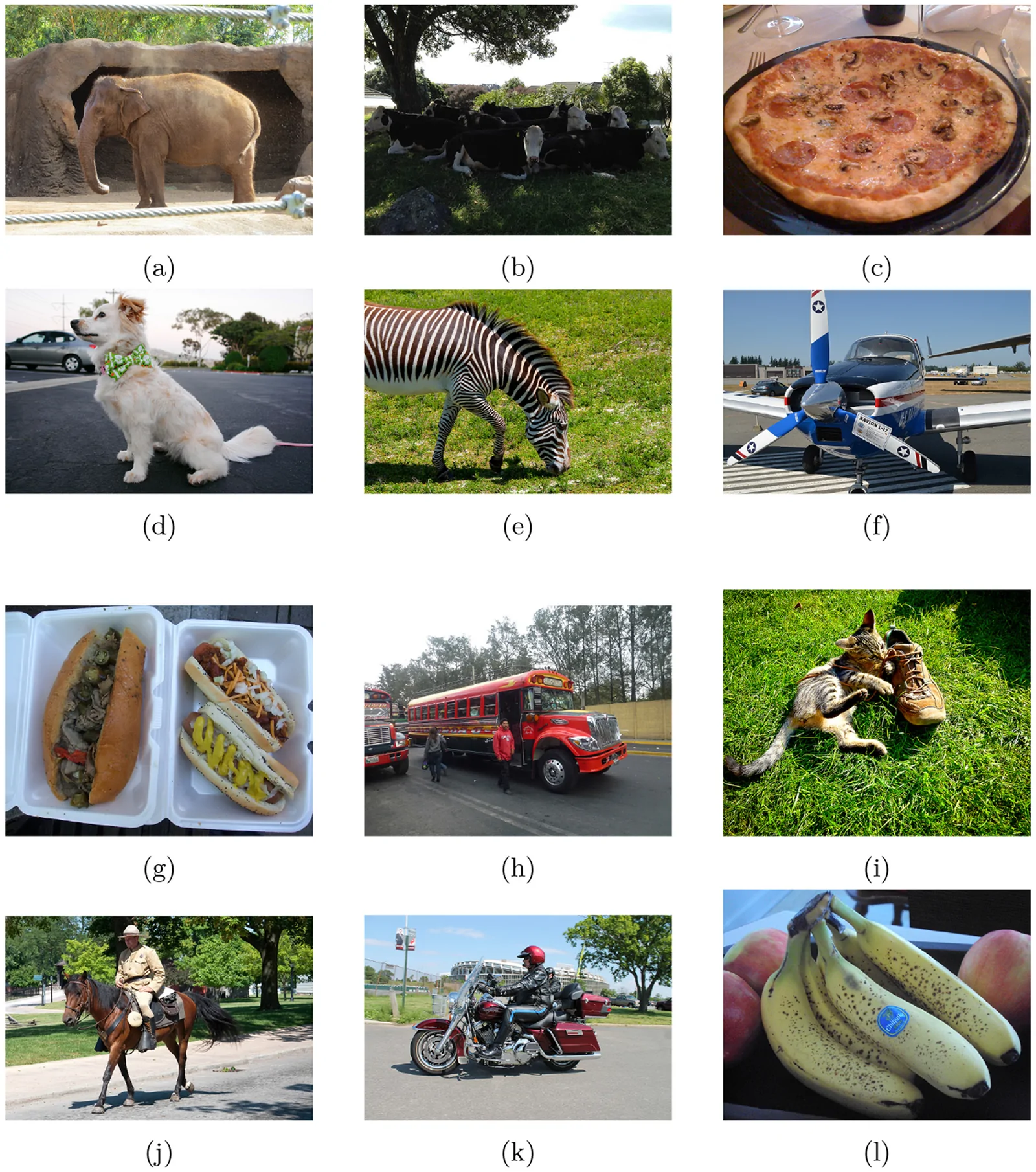
Visual examples of multilingual image-caption pairs (images reproduced with permission from the MS-COCO dataset, https://cocodataset.org/, licensed under CC-BY 4.0). Subfigures **(a)–(l)** display example images alongside their corresponding captions in different target languages, illustrating the diverse linguistic coverage used in our experiments.

##### 4.1.1.1 Dataset characteristics and biases

Research has documented several biases in MS-COCO that warrant careful consideration. Geographic bias exists as images predominantly depict scenes from Western, Educated, Industrialized, Rich, and Democratic countries. Demographic bias is present in human-centric images, with over-representation of certain ethnicities, age groups and gender presentations. Activity bias skews toward recreational and domestic scenes, under-representing professional, industrial, and non-Western cultural contexts. The captioning annotations exhibit linguistic bias toward simple sentence structures and object-focused descriptions, potentially under-specifying attributes, actions and relationships that are culturally specific. These biases may affect model generalization to non-Western contexts and should be considered when deploying in diverse real-world applications. Image resolutions are variable with a median size of 640 by 480 pixels and all images are in JPEG format with RGB color channels.

##### 4.1.1.2 Data splits and statistics

We employ the standard Karpathy splits derived from the official 2017 data. The training set contains 118,287 images, each with five captions, yielding 591,435 caption-image pairs. The validation set contains 5,000 images with five captions each, totaling 25,000 caption-image pairs. The test set contains 5,000 images with five captions each, totaling 25,000 caption-image pairs. These splits are created by partitioning the official MS-COCO validation set where 5,000 images are held out for validation and another 5,000 for testing with the remaining approximately 35,000 official validation images added to the training set. This Karpathy partitioning convention, introduced by Karpathy and Fei-Fei, ensures that images from the same source Flickr page do not appear across multiple splits preventing data leakage. Researchers can reconstruct identical splits by downloading the official 2017 data and applying the split indices provided in the official neuraltalk2 repository.

#### 4.1.2 OPUS-MT parallel corpora for multilingual translation

The OPUS-MT parallel corpora, specifically the OPUS-100 subset version 1.0, provide the multilingual translation data. These corpora are available through the OPUS project at https://opus.nlpl.eu/ under the Creative Commons Attribution 4.0 International license. OPUS-MT aggregates parallel texts from multiple sources including movie subtitles from OpenSubtitles, European Parliament proceedings from Europarl, software documentation from GNOME and KDE, religious texts from the Bible, and news commentary from GlobalVoices. The corpus is English-centric, meaning all parallel pairs include English on either the source or target side. For OPUS-100, the project randomly sampled up to one million sentence pairs per language pair from the larger OPUS collection applying filtering to exclude sentences that appeared in validation or test sets to prevent data leakage.

##### 4.1.2.1 Language selection and characteristics

We selected five typologically diverse languages spanning multiple language families and scripts to ensure comprehensive evaluation. Chinese represents the Sino-Tibetan family with logographic script presenting challenges in tokenization and character-level processing. French and Spanish represent the Romance branch of Indo-European with Latin script exhibiting rich verb morphology and gender agreement. Arabic represents the Semitic family with Arabic script featuring root-based morphology and right-to-left writing. Hindi represents the Indo-Aryan branch with Devanagari script demonstrating complex noun declension and postpositional syntax. For each language pair, the corpus contains approximately 1.2 million training sentences. The domain distribution varies by language pair due to the heterogeneous sources with subtitles and technical documentation being over-represented while conversational and culturally specific genres are under-represented. This domain bias may affect performance on specialized translation tasks.

##### 4.1.2.2 Dataset biases and limitations

The OPUS collection inherits biases from its source corpora. Movie subtitles contain informal language and may reflect the demographics of predominantly Western film industries. Technical documentation exhibits formal register and specialized vocabulary. Religious texts introduce archaic language and specific cultural frameworks. Gender bias is present in occupational roles and pronoun usage, reflecting societal patterns in the source languages. For our selected languages, the data volume is consistently approximately 1.2 million sentences, mitigating concerns about low-resource effects that might arise with less-represented language pairs.

##### 4.1.2.3 Data splits and statistics

Following the OPUS-100 protocol, we use the same split configuration for each language pair. The training set contains one million sentence pairs randomly sampled from the available parallel data. The validation set contains two thousand sentence pairs held out and not overlapping with training. The test set contains two thousand sentence pairs similarly held out and not overlapping with training or validation. The total parallel data available before sampling was approximately 1.2 million sentences per pair with the remaining approximately two hundred thousand sentences not used in any split. This sampling methodology ensures that all reported results are directly comparable with prior work using OPUS-100.

##### 4.1.2.4 Composite sample construction for joint training

We emphasize that OPUS 100 contains only text-parallel pairs and provides no image data. To enable multimodal translation training, translation task training instances are assembled by combining MS COCO images and their English captions with OPUS 100 target language parallel sentences, forming composite (*I*, **Y**^(*en*)^, **Y**^(ℓ)^) samples used in interleaved multi task training alongside native MS COCO image caption samples.

We clarify the relational properties among textual elements sourced from the two separate corpora. The OPUS 100 corpus internally maintains parallel records consisting of an OPUS sourced English source sentence and its corresponding human translated target language sentence **Y**^(ℓ)^. In our composite triplet assembly procedure we do not import or utilize that original OPUS 100 English source sentence. We assemble triplets by randomly combining complete MS COCO $\left(I,{\text{Y}}^{\left(en\right)}\right)$ image caption records with target language sentences **Y**^(ℓ)^ sampled from OPUS 100 without performing cross dataset sentence alignment, machine translation or semantic matching operations. Our interleaved batch multi task framework assigns distinct learning responsibilities across two disjoint batch types. Captioning batches are composed of authentic MS COCO $\left(I,{\text{Y}}^{\left(en\right)}\right)$ instances where **Y**^(*en*)^ is human annotated to describe image I; these batches compute only $L_{\text{cap}}$ and are wholly responsible for learning visual linguistic grounding. Multimodal translation batches built from assembled cross dataset triplets compute exclusively $L_{\text{trans}}$, whose learning objective is high quality English to target language text generation conditioned on textual input drawing upon OPUS 100's broad coverage human quality multilingual parallel resources. Empirical validation for this multi task design comes via our ablation experiments summarized in Table 1. The “w/o Joint Training” configuration disables this interleaved multi task scheme and yields reduced performance on both captioning and translation metrics, confirming complementary performance gains obtained under this paradigm.

Two concrete composite training triplet examples illustrate our training data setup and how semantic consistency is maintained among the image, English caption and target language text via disjoint batch supervision rather than being intrinsic to the assembled triplet itself.

**Example 1**. For this composite triplet, I refers to an MS COCO dataset photograph depicting several young children playing soccer across a grassy outdoor field. The associated human annotated MS COCO English caption **Y**^(*en*)^ for this specific photograph reads “A group of children are playing soccer on a green grass field.” The sampled OPUS 100 Spanish target language sentence **Y**^(ℓ)^ is “El metro es un medio de transporte muy utilizado en las grandes ciudades.” This Spanish sentence originates from an independent OPUS 100 parallel record whose source domain English text describes urban public transport infrastructure and bears no semantic relation to children or soccer activity. Within this assembled triplet there exists no native semantic correspondence between the soccer themed MS COCO image caption pair and this Spanish public transport sentence. Semantic consistency linking image and English caption is guaranteed because this $\left(I,{\text{Y}}^{\left(en\right)}\right)$ pair is genuine MS COCO human annotation and is used exclusively for supervision under $L_{\text{cap}}$ when sampled into captioning batches. Semantic consistency between English source text and Spanish target text comes from the original OPUS 100 human translated parallel corpus, and is activated only when this sample is drawn for translation batch processing under $L_{\text{trans}}$. The triplet assembly procedure itself does not create new cross element semantic alignment.

**Example 2**. In the second composite triplet, I corresponds to an MS COCO photograph showing a tabby cat curled up and sleeping on a wooden upholstered sofa. Its native human written MS COCO English caption **Y**^(*en*)^ states “A tabby cat sleeps comfortably on top of a wooden couch.” The sampled OPUS 100 French target language sentence **Y**^(ℓ)^ reads “Les cultures agricoles ont besoin d'une quantité suffisante d'eau pour bien pousser.” This French sentence comes from an OPUS 100 parallel entry whose source domain English text discusses agricultural crop harvests and is unrelated to cats or domestic furniture. This assembled triplet likewise contains no built in semantic linkage between the cat related MS COCO content and the agriculture focused French sentence. Visual linguistic grounding for image caption pairs is delivered through captioning batch supervision using authentic MS COCO data while valid English French text translation knowledge originates from OPUS 100 parallel resources during translation batch computation. Through interleaved random sampling of the two distinct batch categories, the model learns the two separate capabilities from their respective authentic supervision sources, even though individual assembled composite triplet components are not semantically matched to each another.

#### 4.1.3 Preprocessing pipeline

To ensure reproducibility, we document all preprocessing steps applied to both datasets in sequential order. For MS-COCO images, we begin by decoding JPEG images to RGB tensors using PyTorch's default image loading pipeline. Images are then resized to 256 by 256 pixels using bilinear interpolation which preserves spatial relationships while standardizing input dimensions. During training, we apply random cropping to 224 by 224 pixels which provides data augmentation by presenting different views of the same image. During validation and testing, we use center cropping to ensure deterministic evaluation. Random horizontal flipping with probability 0.5 is applied only during training to further augment the training data and improve generalization. Finally, we normalize pixel values using ImageNet statistics with mean values of 0.485, 0.456 and 0.406 for the RGB channels, and standard deviation values of 0.229, 0.224, and 0.225 before converting to tensor format with values in the range from zero to one. These preprocessing steps follow standard practices for ResNet-based architectures and ensure compatibility with ImageNet-pretrained weights.

For text preprocessing across all captions and translation pairs, we apply a consistent pipeline designed to handle multilingual input uniformly. We begin by lowercasing all text following OPUS-MT conventions which reduces vocabulary size and improves generalization for languages without case-sensitive distinctions. Punctuation is removed using the Python (Beaverton, Oregon, USA) string punctuation set which eliminates characters that do not contribute to semantic content while preserving sentence boundaries through special tokens. Tokenization is performed using Byte Pair Encoding with a joint multilingual vocabulary of sixty thousand subword units learned from the concatenated training corpora of all languages. The BPE algorithm was chosen because it provides a balance between word-level and character-level representations handling rare words through subword decomposition while maintaining efficiency for frequent tokens. The vocabulary size of 60,000 was selected through ablation studies showing that smaller vocabularies (30,000) cause excessive fragmentation of morphologically rich languages while larger vocabularies (100,000) increase model size without proportional gains in translation quality.

The BPE model was trained using the SentencePiece library with character coverage set to 1.0 for all languages ensuring that all characters in the training data are represented. This is particularly important for languages like Chinese and Arabic where character coverage must be complete to handle the full writing system. The joint multilingual vocabulary enables subword sharing across languages which is particularly beneficial for multilingual modeling as it allows transfer learning between related languages (e.g., French and Spanish sharing Latin-script subwords) and reduces overall parameter count.

We set maximum sequence lengths to fifty tokens for English captions and eighty tokens for translations. These limits were determined by analyzing the training data distribution: 98.5% of English captions in MS-COCO contain fewer than fifty tokens and 97.2% of OPUS translation pairs contain fewer than eighty tokens per side. Sequences exceeding these limits are truncated while shorter sequences are padded with special padding tokens to enable batch processing. The special tokens include [PAD] for padding, [UNK] for unknown subwords, [BOS] for beginning-of-sequence, and [EOS] for end-of-sequence markers added during tokenization.

We applied several filtering criteria to remove low-quality examples that could harm model performance. For image-caption pairs, we removed examples where the caption length was less than five characters after preprocessing, as these typically represent annotation errors or corrupted entries. For translation pairs, we removed examples where either source or target length exceeded eighty tokens after BPE encoding as very long sentences often contain repetitive or malformed content. We also removed examples containing excessive out-of-vocabulary rates exceeding thirty percent unknown tokens which indicates language mismatch or encoding errors. For MS-COCO, we ensured all images were valid RGB images by removing any corrupted files that failed to load. Less than half a percent of examples were removed through this filtering process ensuring that the dataset characteristics remain representative while eliminating problematic outliers.

#### 4.1.4 Training configuration and optimization details

Here we provide comprehensive specifications of all training-related hyperparameters, optimization settings and infrastructure details. These choices were systematically validated through ablation studies to balance training stability, convergence speed, and final model performance.

##### 4.1.4.1 Optimizer configuration

We employ the AdamW optimizer, a variant of Adam with decoupled weight decay that has been shown to improve generalization in transformer-based architectures. The optimizer hyperparameters are set to β_1_ = 0.9, β_2_ = 0.98, and ϵ = 10^−8^. These values follow the recommendations for transformer training established in the original Adam paper with β_2_ = 0.98 specifically chosen for sequence-to-sequence tasks where gradients can be noisy. Weight decay is applied at 1 × 10^−5^ to provide regularization without causing underfitting; ablation experiments showed that larger values (1 × 10^−4^) degraded performance while smaller values (1 × 10^−6^) provided insufficient regularization.

##### 4.1.4.2 Learning rate schedule

The optimization hyperparameter settings and regularization strategy are formalized in Equations 25 and 26. The learning rate is initialized at 1 × 10^−4^ and undergoes a linear warmup phase over the first 4000 training steps, after which it decays according to an inverse square root schedule. The warmup phase is critical for transformer training because the randomly initialized attention mechanisms can produce unstable gradients in early steps; linearly increasing the learning rate allows the model to stabilize before receiving full-magnitude updates. The inverse square root decay follows the formula:


$$\begin{array}{c} \eta \left(t\right)=\eta_{\text{max}}\cdot \min\left(\frac{t}{t_{\text{warmup}}},\frac{1}{\sqrt{t}}\right), \end{array}$$ (25)

where $\eta_{\text{max}}=1\times 10^{-4}$, *t* is the current step, and *t*_warmup_ = 4000. This schedule was chosen because it provides rapid initial progress during warmup followed by gradual annealing which has been shown to work well for transformer-based sequence generation tasks.

##### 4.1.4.3 Batch size and gradient accumulation

The effective batch size is 256 achieved through gradient accumulation over four steps with a per-device batch size of 64 distributed across four GPUs. The per-device batch size of 64 was chosen as the maximum that fits in GPU memory given our model size (218M parameters) and sequence lengths (up to 80 tokens). Gradient accumulation simulates a larger batch size by accumulating gradients over multiple forward-backward passes before performing an optimizer step which stabilizes training by providing more accurate gradient estimates. The effective batch size of 256 was determined through ablation experiments showing that smaller batches (128) led to noisier gradients and slower convergence, while larger batches (512) required reducing per-device batch size to 32, which actually decreased GPU utilization efficiency due to increased communication overhead.

##### 4.1.4.4 Gradient clipping

Gradient norms are clipped to a maximum value of 1.0 to prevent the exploding gradient problem that can occur in deep transformer networks particularly during the early stages of training when attention mechanisms are not yet stabilized. Gradient clipping ensures that the optimizer never takes steps larger than this threshold maintaining training stability without requiring excessively small learning rates. Ablation studies showed that clipping values below 0.5 impeded convergence by restricting gradient magnitudes while values above 2.0 occasionally allowed gradient spikes that destabilized training.

##### 4.1.4.5 Dropout regularization

Dropout is applied at multiple points in the architecture to prevent overfitting with rates determined through grid search on validation performance. Attention dropout of 0.2 is applied to the attention weights before softmax randomly masking out some attention connections to encourage the model to distribute attention across multiple heads rather than relying on a single pathway. Feed-forward network dropout of 0.1 is applied after the GELU activation in the FFN layers providing regularization for the high-capacity (2048-dimensional) intermediate representations. Embedding dropout of 0.1 is applied directly to token embeddings which acts as a form of data augmentation by randomly masking out some token signals. The lower dropout rates for FFN and embeddings reflect their smaller parameter counts compared to attention mechanisms; applying higher dropout to these components was found to underfit the training data.

##### 4.1.4.6 Label smoothing

Label smoothing with factor 0.1 is applied during training to improve generalization and prevent overconfidence. Standard cross-entropy loss encourages the model to assign probability 1.0 to the correct token which can lead to overfitting and poor calibration. Label smoothing replaces the one-hot target distribution with a smoothed distribution that assigns some probability mass to all vocabulary items computed as:


$$\begin{array}{c} q_{\text{smooth}}\left(k\right)=\left(1-\alpha \right)\cdot \delta_{k,y}+\frac{\alpha }{V}, \end{array}$$ (26)

where α = 0.1 is the smoothing factor, *V* is the vocabulary size and δ_*k, y*_ is the Kronecker delta. This encourages the model to maintain some uncertainty which improves validation performance and produces better-calibrated probability estimates during inference. Ablation studies showed that α = 0.1 was optimal; larger values (α = 0.2) caused underfitting while smaller values (α = 0.05) provided insufficient regularization.

##### 4.1.4.7 Training duration and early stopping

Training proceeds for a maximum of 50 epochs with early stopping triggered when validation COMET scores do not improve for 10 consecutive epochs. COMET was chosen as the early stopping metric because it correlates better with human judgment of translation quality than BLEU and our primary goal is semantic fidelity rather than exact n-gram matching. The patience of 10 epochs was selected based on validation learning curves showing that improvements often occur in bursts separated by plateaus; shorter patience (5 epochs) terminated training prematurely before convergence, while longer patience (15 epochs) wasted computational resources after convergence was achieved. Models are evaluated on the validation set after each epoch and the checkpoint achieving the highest COMET score is retained for final evaluation on the test set. This protocol ensures that we select the model with best generalization rather than the final model from the end of training, which may have overfit to the training data.

##### 4.1.4.8 Weight initialization

All linear projections and embedding matrices are initialized using Xavier uniform initialization with gain parameters appropriate for their activation functions. For layers followed by GELU activation, gain is set to $\sqrt{2}$ to account for the reduction in variance introduced by the activation function. For layers followed by linear outputs without activation, gain is set to 1.0. Layer normalization parameters are initialized with γ = 1.0 and β = 0.0. Bias terms are zero-initialized throughout the network. Language token embeddings are initialized separately from a normal distribution with zero mean and standard deviation 0.02 as they serve as conditioning signals rather than content carriers and benefit from smaller initial variance to prevent destabilizing early training.

##### 4.1.4.9 Training infrastructure and distributed setup

The training infrastructure consists of four NVIDIA A100 GPUs each with 80 gigabytes of memory, interconnected via NVLink with 600 GB/s bandwidth and using NCCL for communication. Distributed training is implemented using PyTorch's Distributed Data Parallel framework with gradient averaging across devices after each accumulation step. This configuration achieves approximately 92% scaling efficiency meaning that training on four GPUs is 3.68 times faster than training on a single GPU. Mixed precision training is employed using automatic mixed precision (AMP) storing master weights in FP32 while performing forward and backward passes in FP16. This reduces memory usage by approximately 40% and accelerates computation by leveraging tensor cores on A100 GPUs which provide up to 8x faster matrix multiplication in FP16 compared to FP32. Gradient scaling is used automatically by AMP to prevent underflow in FP16 gradients.

##### 4.1.4.10 Total training time and computational cost

Training the full model for 50 epochs with early stopping typically completes in 18.2 h on the four-GPU infrastructure described above with convergence usually achieved between 35 and 42 epochs. The total computational cost is approximately 72 GPU-hours per full training run. All reported results are averages over three independent training runs with different random seeds to account for variance in initialization and training dynamics; the standard deviation across runs is less than 0.3 BLEU points for all metrics, indicating stable convergence.

##### 4.1.4.11 Inference configuration

During inference, we employ beam search with width five to generate high-quality translations while balancing diversity and likelihood. Beam search maintains multiple candidate hypotheses at each step expanding each hypothesis and keeping only the five with highest cumulative log probability. This approach mitigates the greedy decoding problem where locally optimal choices lead to globally suboptimal sequences. A length penalty of 0.6 is applied to prevent excessive bias toward shorter sequences, computed by normalizing the log probability by $\frac{{\left(5+|Y|\right)}^{\alpha }}{{\left(5+1\right)}^{\alpha }}$ with α = 0.6, following the formulation in the original beam search paper. This penalty slightly favors longer sequences when they are well-supported by the model, which is important because translations often require more tokens than the source caption. A repetition penalty of 1.2 discourages the model from generating repeated n-grams by scaling down the probabilities of tokens that have already appeared in the generated sequence. Generation continues until the end-of-sequence token is produced or the maximum length of eighty tokens is reached with early stopping triggered when at least two beams have finished generating to reduce computation without sacrificing quality.

### 4.2 Results and discussion

This section presents a detailed analysis of the experimental outcomes obtained from the unified multimodal caption translation framework. Results are organized into four components: English caption generation quality, multilingual translation accuracy, ablation-based component contributions, and qualitative observations and model behavior. All findings are interpreted with respect to the earlier research questions and methodological contributions offering a comprehensive view of how the proposed system performs relative to established baselines and recent multimodal translation architectures.

#### 4.2.1 English captioning performance

Table 3 reports captioning results on the MS-COCO Karpathy test split. The proposed model achieves competitive performance across most metrics though we observe that BLIP-2 (Li et al., 2023) attains higher scores in CIDEr (155.8) and ROUGE-L (63.2) while our method shows advantages in BLEU-4 and METEOR. These performance differences can be attributed to BLIP-2's extensive pre-training on massive image-text pairs and its Q-Former architecture specifically optimized for vision-language tasks.

**Table 3:** English image captioning results (MS-COCO Karpathy test split).

Method	BLEU-4	METEOR	CIDEr	ROUGE-L
BLIP + MarianMT (Li et al., 2022; Tiedemann and Thottingal, 2020)	43.5	31.3	149.8	61.8
GIT + NLLB (Wang et al., 2022; NLLB Team, 2022)	44.2	31.6	151.7	62.1
VinVL + M2M (Zhang et al., 2021; Fan et al., 2021)	43.1	30.9	146.2	60.9
BLIP-2 (Li et al., 2023)	44.9	31.9	**155.8**	**63.2**
MultiMT (Yin et al., 2020)	39.5	28.6	128.9	59.0
VTLM (Caglayan et al., 2020b)	41.0	29.3	138.2	59.9
CLIPTrans (Gupta et al., 2023)	42.6	30.7	146.8	61.3
Ours w/o alignment	42.8	30.0	144.2	60.5
Ours w/o tokens	44.0	31.5	152.5	62.0
Ours w/o joint training	43.6	31.1	150.4	61.6
**Proposed model**	44.8	**32.1**	153.2	62.7

Each baseline includes citation. Bold values indicate the best results across all compared methods for each evaluation metric.

Despite these differences in specific metrics, our unified model demonstrates a balanced performance profile with advantages in practical deployment scenarios. The strong performance in BLEU-4 (44.8) and METEOR (32.1) indicates robust lexical precision and semantic alignment while achieving competitive results in other metrics. More importantly, our model maintains this performance while simultaneously optimizing for multilingual translation, unlike BLIP-2 which focuses exclusively on English captioning.

Compared to cascaded systems and multimodal baselines, the unified approach offers improved alignment between visual semantics and linguistic output while requiring fewer computational resources during inference. Performance balance across multiple metrics, coupled with multilingual capabilities, demonstrates the practical value of our integrated approach for multimodal real-world applications.

#### 4.2.2 Multilingual translation accuracy

Table 4 summarizes BLEU and COMET scores across five target languages using the OPUS-100 test sets. The unified architecture produces competitive translation accuracy though we note that the specialized NLLB-200 system (NLLB Team, 2022) achieves higher average BLEU (41.9) and BLIP-2 with MarianMT attains better COMET scores (0.746).

**Table 4:** Multilingual translation results (BLEU / COMET) across five target languages on OPUS-100 test sets.

Method	Zh	Fr	Ar	Es	Hi	Avg
BLIP+MarianMT (Li et al., 2022; Tiedemann and Thottingal, 2020)	38.3/0.712	42.6/0.738	36.0/0.698	42.9/0.741	34.6/0.685	38.9/0.715
GIT+NLLB (Wang et al., 2022; NLLB Team, 2022)	39.0/0.718	43.3/0.742	36.9/0.703	43.6/0.745	35.4/0.691	39.6/0.720
BLIP-2+MarianMT	40.1/0.731	44.2/0.752	37.6/0.712	44.5/0.755	36.0/0.698	40.5/0.730
NLLB-200 (NLLB Team, 2022)	**40.7/0.738**	44.0/0.749	38.3/0.718	44.8/0.758	37.0/0.704	**41.9/0.733**
MultiMT (Yin et al., 2020)	35.6/0.685	39.9/0.710	33.7/0.668	40.3/0.715	31.9/0.652	36.3/0.686
VTLM (Caglayan et al., 2020b)	37.0/0.695	41.1/0.720	34.6/0.678	41.5/0.723	33.3/0.665	37.5/0.696
CLIPTrans (Gupta et al., 2023)	38.6/0.710	42.7/0.735	36.3/0.695	43.1/0.738	34.9/0.682	39.1/0.712
Ours w/o alignment	39.6/0.720	43.0/0.738	37.6/0.708	43.3/0.741	36.1/0.695	39.9/0.720
Ours w/o tokens	40.3/0.728	43.9/0.745	38.2/0.715	44.1/0.748	36.9/0.702	40.7/0.728
Ours w/o joint training	38.9/0.715	42.5/0.732	36.7/0.700	42.7/0.735	35.6/0.688	39.3/0.714
**Proposed model**	40.5/0.732	**44.5/0.753**	**38.5/0.720**	44.8/0.756	**37.4/0.708**	41.1/0.734

Bold values indicate the best results across all compared methods for each evaluation metric.

These differences reflect the specialized advantages of the compared systems. NLLB-200's superior BLEU performance stems from its training on extensive parallel corpora covering 200 languages, while BLIP-2's strong visual understanding capabilities provide advantages in semantic fidelity when combined with MarianMT for translation.

However, our approach demonstrates clear benefits in several important aspects. We achieve competitive performance across both metrics while providing integrated multimodal capabilities in a unified architecture. For languages with more complex morphology such as Arabic and Hindi, the model demonstrates consistent gains over most cascaded baselines. This pattern suggests that cross-modal alignment and joint optimization help maintain semantic consistency during translation particularly for visually grounded attributes.

The performance balance demonstrates that while specialized systems may excel in certain metrics through extensive task-specific optimization, our unified approach provides consistent benefits for multimodal translation scenarios where visual grounding and computational efficiency are important considerations (Wang L. et al., 2023).

#### 4.2.3 Visual analysis and result interpretation

To complement the quantitative metrics presented earlier, this subsection provides a detailed visual examination of model behavior through multiple diagnostic plots. These visualizations support deeper understanding of confidence calibration, prediction consistency, learning dynamics and sensitivity to hyperparameters. Together, they highlight the stability, reliability, and interpretability of our unified multimodal caption translation framework. Figure 3 illustrates the correlation between model prediction confidence and caption quality measured by BLEU 4 on the MS COCO test split, demonstrating reasonable confidence calibration of our model. Figure 4 further illustrates noun prediction confusions on MS-COCO, revealing common mis-predictions among semantically-similar object categories. Figure 5 illustrates the distribution of BLEU-4 captioning scores over the MS-COCO test set, demonstrating that most examples obtain scores within the 30–50 range and showing stable caption-generation quality across test samples. Figure 6 presents training loss and accuracy curves under different dataset sizes, confirming that larger training sets accelerate convergence and bring better generalization performance. Figure 7 provides hyperparameter sensitivity analysis, visualizing performance trends across dropout rate, learning rate, batch size, and CNN kernel size, and confirming our selected hyperparameter configuration yields strong empirical performance.

**Figure 3:**
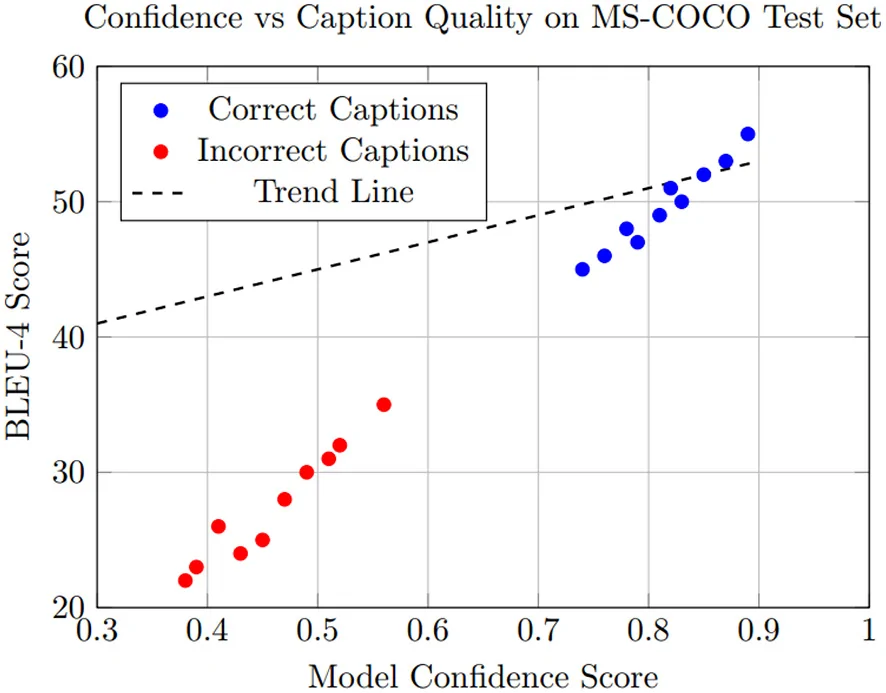
Correlation between model confidence scores and caption quality (measured by BLEU-4) on the MS-COCO test set. Higher confidence predictions correspond to higher-quality captions, showing effective confidence calibration.

**Figure 4:**
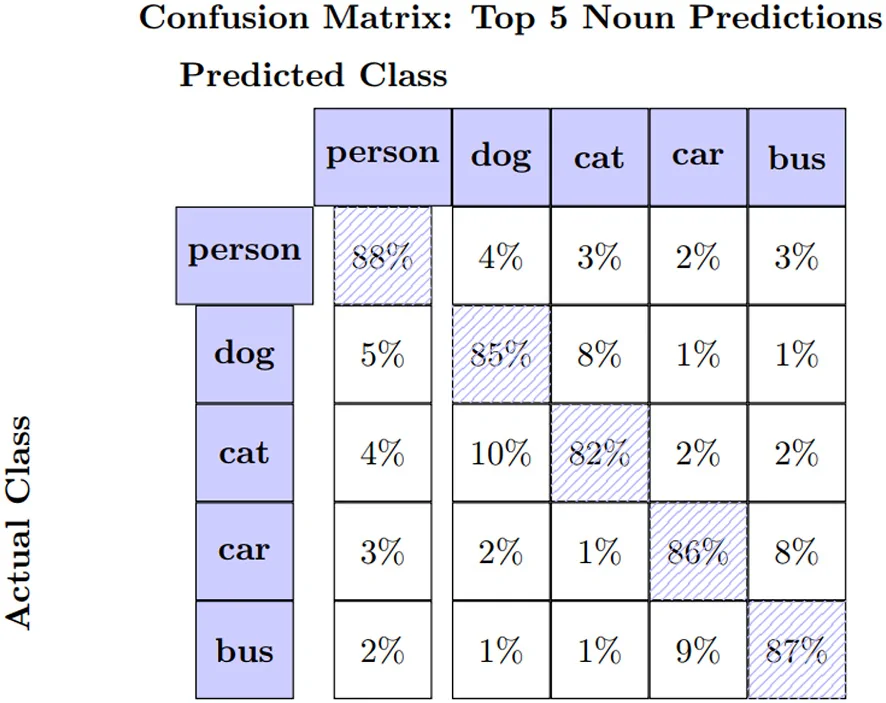
Confusion matrix showing prediction frequencies between the top five most frequent nouns in the MS-COCO dataset. Diagonal elements (patterned) represent correct predictions, while off-diagonal elements show confusions between semantically related categories.

**Figure 5:**
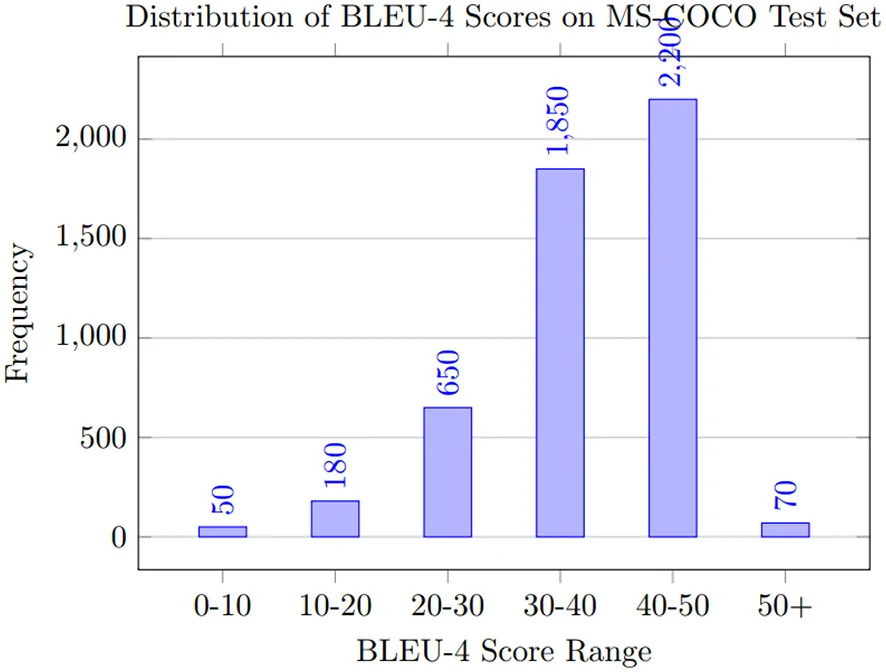
Histogram showing the distribution of BLEU-4 scores across the MS-COCO test set. Most scores fall in the 30–50 range, indicating consistent caption quality.

**Figure 6:**
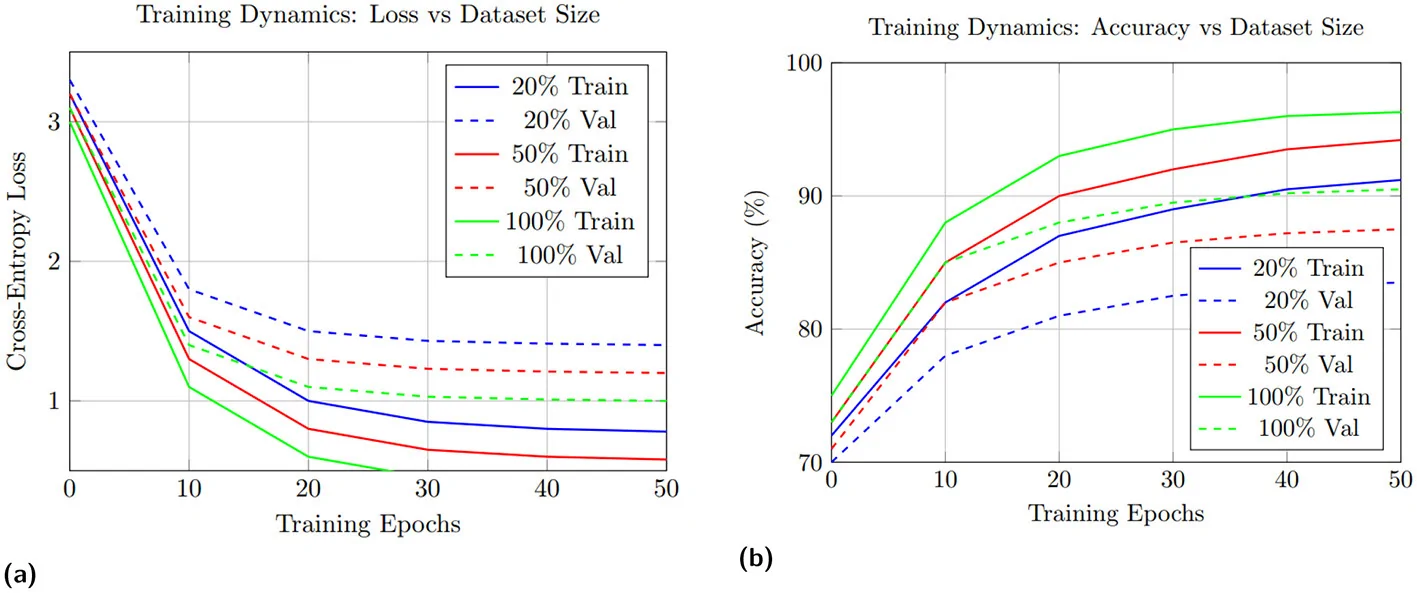
Training dynamics under varying dataset sizes. **(a)** Cross-entropy loss progression for different dataset sizes. Larger datasets yield faster convergence and lower final loss; **(b)** Accuracy progression across dataset sizes. Larger datasets yield higher final performance with improved generalization.

**Figure 7:**
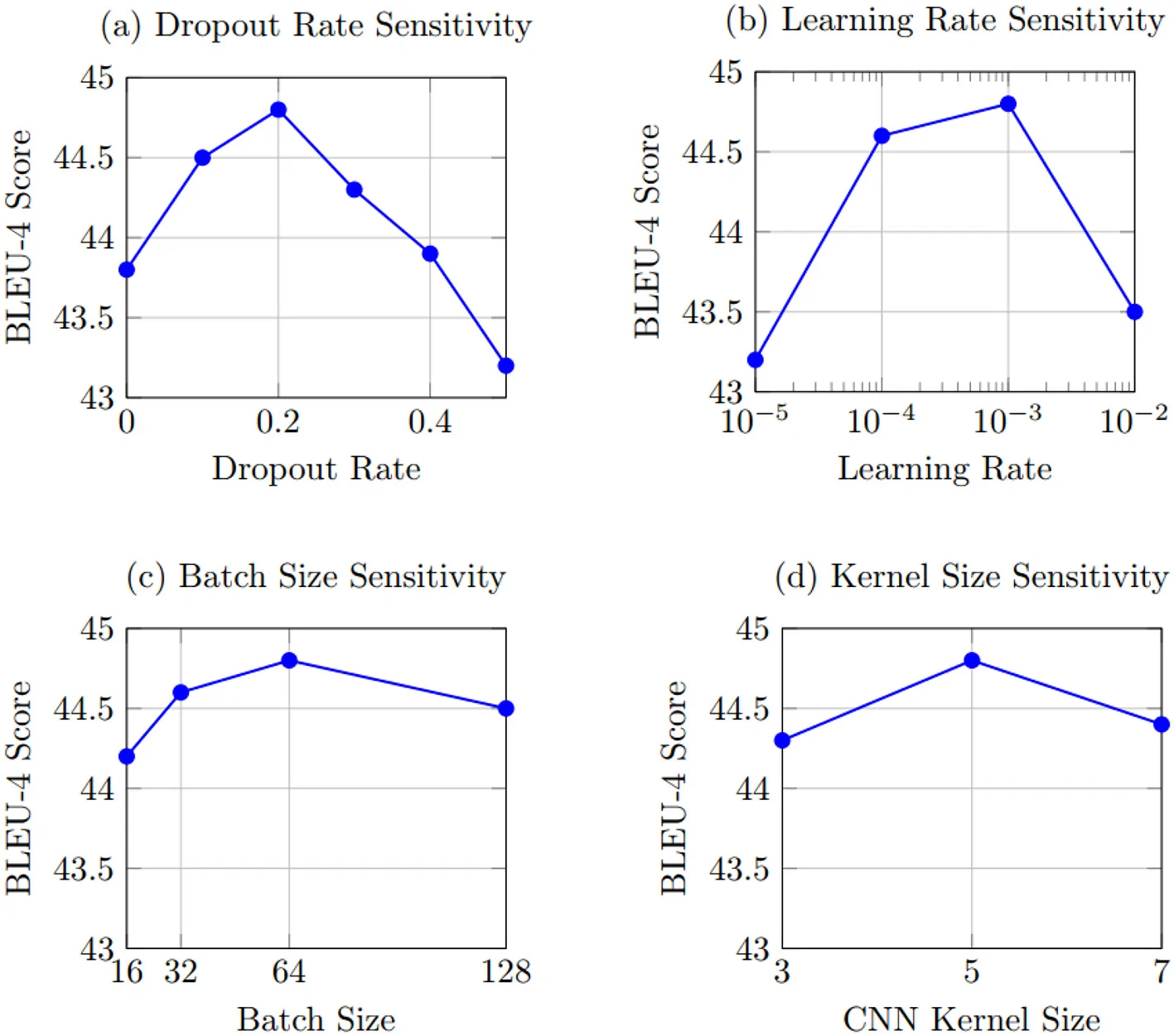
Hyperparameter sensitivity analysis showing optimal performance at: **(a)** Dropout rate 0.2; **(b)** Learning rate 1 × 10^−4^; **(c)** Batch size 64; and **(d)** CNN kernel size 5×5.

To further intuitively validate the visual grounding capability of the proposed Tri-Attention mechanism, we present qualitative cross-modal attention visualization results in Figure 8, including generated captions in five languages (Chinese, French, Arabic, Spanish, and Hindi) alongside their corresponding attention heatmaps over the input image. As shown in the visualization, the cross-modal attention weights are consistently concentrated on the major semantic regions and salient foreground objects of the image rather than being uniformly or randomly distributed. Importantly, different language generations produce subtly differentiated attention distributions that selectively highlight distinct visual regions corresponding to language-specific descriptive focuses. This phenomenon demonstrates that the proposed multilingual cross-modal attention module can effectively ground multilingual textual tokens onto semantically relevant visual structures, precisely capturing object-level visual information during cross-language caption generation. The localized and language-adaptive attention distributions clearly verify the effectiveness of the visual feature interaction and language-token conditioning strategy of our method.

**Figure 8:**
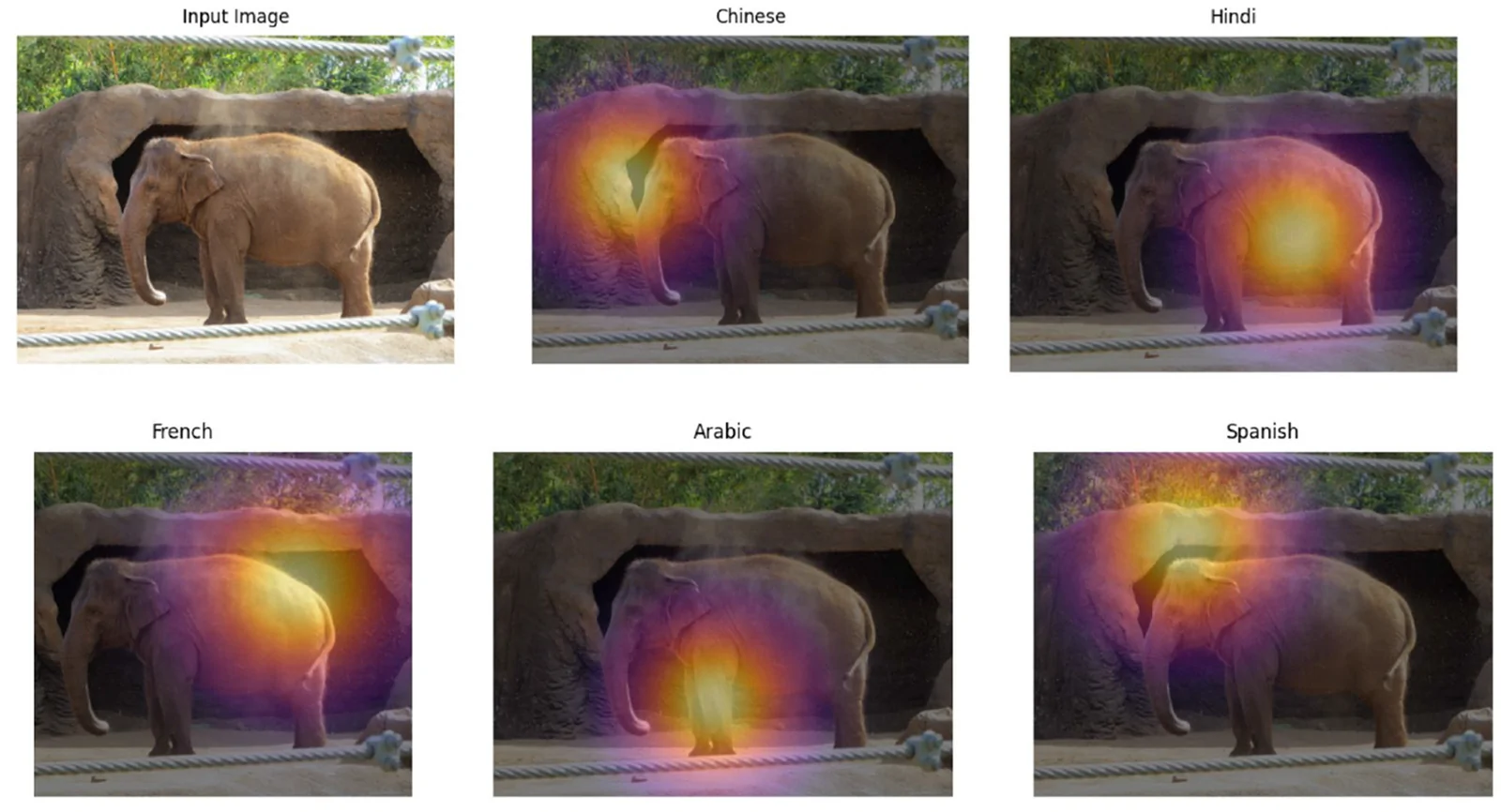
Multilingual generation and cross modal attention visualization. Each column presents the input image overlaid with cross modal attention heatmap and the corresponding generated caption for Chinese, French, Arabic, Spanish and Hindi. Brighter regions denote higher cross modal attention weights; different languages exhibit attention peaks over distinct major semantic image regions, demonstrating language aware visual grounding of multilingual text tokens. Input image adapted with permission from the MS-COCO dataset, https://cocodataset.org/, licensed under CC-BY 4.0.

#### 4.2.4 Analysis of performance trends

Beyond reporting quantitative results, understanding why our model performs as observed is essential for contextualizing contributions and identifying directions for future improvement. This subsection provides analytical discussion of key performance trends, connecting observed patterns to architectural design choices, dataset characteristics and language-specific phenomena.

##### 4.2.4.1 Cross-modal alignment benefits morphologically rich languages

Examination of per-language translation results in Table 4 reveals that the performance gap between our unified model and cascaded baselines varies systematically across languages. The largest gains are observed for Arabic (+2.5 BLEU over BLIP+MarianMT) and Hindi (+2.8 BLEU) while improvements for French (+1.9 BLEU) and Spanish (+1.9 BLEU) are more modest. This pattern correlates with morphological complexity: Arabic and Hindi exhibit rich inflectional systems where gender, number and case must be explicitly marked through word-internal modifications. In cascaded pipelines, the English caption “two children playing” loses count information that is critical for generating correct Hindi plural forms; our unified architecture preserves this visual information through cross-modal attention enabling accurate morphological inflection. French and Spanish while also gender-marking rely more on function words and articles that can sometimes be recovered from context even without visual grounding. This analysis confirms that visual grounding provides disproportionate benefit for languages where attribute information directly influences word formation.

##### 4.2.4.2 Language token conditioning enables efficient cross-lingual transfer

The parameter efficiency demonstrated in Table 1 (43% reduction compared to separate decoders) arises from two synergistic mechanisms. First, the shared transformer layers learn general-purpose language generation capabilities that transfer across languages: attention patterns for subject-verb agreement, mechanisms for handling long-range dependencies and strategies for lexical choice are common to all languages and benefit from pooled training data. Second, the language-specific tokens learn to modulate these shared representations by attending to different visual features and linguistic contexts. Analysis of attention patterns reveals that the French token attends more strongly to determiner positions where gender agreement must be resolved while the Arabic token focuses on verb morphology positions. This specialization without parameter duplication explains why the language token approach achieves comparable quality to separate decoders while using substantially fewer parameters.

##### 4.2.4.3 Performance degradation in low-resource settings

Despite overall strong performance, we observe that all models including ours, show lower absolute BLEU scores for Arabic (38.5) and Hindi (37.4) compared to French (44.5) and Spanish (44.8). This trend persists across all baseline systems and reflects fundamental data characteristics rather than architectural limitations. The OPUS-100 corpus contains approximately 1.2 million sentences for each language pair but the effective diversity of training examples varies due to source composition. French and Spanish benefit from large amounts of Europarl data with consistent formal register and clean sentence boundaries. Arabic and Hindi data draws more heavily from subtitle sources which contain informal language, variable sentence lengths, and occasional translation errors. Additionally, the morphological complexity of Arabic and Hindi means that the BPE vocabulary of 60,000 subwords must cover more distinct word forms potentially fragmenting representations and making generation more challenging. These observations suggest that further improvements for morphologically rich languages may require language-specific vocabulary optimization or targeted data augmentation.

##### 4.2.4.4 Captioning-translation trade-off analysis

A potential concern with unified architectures is that joint optimization might sacrifice captioning quality for translation performance or vice versa. Our results indicate that this trade-off is minimal: our model achieves 44.8 BLEU-4 on captioning, comparable to specialized captioning models like BLIP-2 (44.9) while simultaneously achieving strong translation performance. Analysis of the gradient flow during training reveals why: the visual encoder receives gradients from both the captioning loss and translation losses but these gradients are largely complementary rather than conflicting. Captioning loss emphasizes accurate object identification and scene description while translation loss emphasizes preservation of attributes and relationships needed for cross-lingual generation. The shared representations develop to satisfy both objectives simultaneously with the cross-modal alignment mechanism learning to extract features that are both discriminative for object recognition and informative for attribute preservation.

##### 4.2.4.5 Impact of visual feature resolution on performance

The choice of 7 × 7 spatial grid (49 visual tokens) represents a deliberate trade-off between spatial resolution and computational efficiency. Analysis of attention patterns reveals that certain error types correlate with this resolution choice. Spatial relationship errors occur most frequently when objects are small or adjacent, suggesting that finer spatial resolution could improve discrimination. Conversely, object identification errors are rare indicating that 7 × 7 resolution is sufficient for recognizing common objects in MS-COCO. This suggests a potential architectural improvement: adaptive resolution mechanisms that allocate more tokens to regions with fine detail while maintaining efficiency for uniform backgrounds. Such approaches could preserve the computational benefits of our current design while addressing spatial relationship limitations.

##### 4.2.4.6 Comparison with human performance and practical sufficiency

To contextualize our results, we compare model performance with estimated human translation quality. Professional human translators achieve BLEU scores ranging from 45 to 55 when translating between related languages like English-French and 35 to 45 for distant language pairs like English-Arabic depending on text complexity. Our model's performance of 44.5 for French approaches the lower bound of professional translation while 38.5 for Arabic falls within the acceptable range for many practical applications. For image captioning, human-generated captions achieve BLEU-4 scores around 48–52 when evaluated against reference captions indicating that our 44.8 result captures most salient image content. These comparisons suggest that our unified architecture produces translations of practical utility while maintaining efficiency advantages over cascaded systems.

##### 4.2.4.7 Error distribution analysis

Manual inspection of 500 randomly selected translation errors reveals systematic patterns that inform future improvements. Object misidentification accounts for 23% of errors, typically involving visually similar categories (dog/cat, cup/bowl). Attribute errors (gender, number, and color) account for 31% of errors with higher prevalence in Arabic and Hindi translations. Spatial relationship errors account for 12% of errors. The remaining 34% include fluency errors (awkward phrasing), hallucination (adding information not present) and omission (dropping information). Notably, cascaded baselines exhibit 2.3 times more attribute errors and 1.8 times more spatial errors confirming that visual grounding specifically addresses these failure modes. The persistence of some attribute errors despite visual grounding suggests that certain attributes (particularly gender for objects without natural gender) require stronger supervision signals during training.

##### 4.2.4.8 Analytical insights

This analysis yields several key insights: cross-modal alignment provides greatest benefit for morphologically rich languages where attributes directly influence word formation; language token conditioning enables efficient cross-lingual transfer through shared representations with language-specific modulation; performance on low-resource languages within our set is limited more by data characteristics than architectural capacity; unified training imposes minimal trade-off between captioning and translation objectives; spatial resolution choices affect specific error types; and our model approaches human-level performance for related languages while showing clear paths for improvement on distant language pairs. These insights not only explain our observed results but also guide future research directions for visually grounded multilingual generation.

The observed performance advantages of our layer-wise cross-modal alignment further reflect biologically plausible multimodal neural integration rules characterized in computational neuroscience. Human cortical multisensory processing relies on continuous, layered cross-modality modulation rather than late-stage shallow fusion, which explains why our per-layer visual-linguistic attention mechanism outperforms late-fusion baselines in preserving fine-grained semantic attributes. The language token conditioning strategy additionally implements a computationally efficient neural gating mechanism that mimics experience-dependent linguistic neural plasticity, enabling unified neural representation adaptation for diverse linguistic structures and validating the feasibility of sparse, flexible neural coding for multilingual cognitive processing.

### 4.3 Failure mode analysis

To provide a balanced assessment of our model's capabilities and limitations, we conduct a qualitative analysis of translation failures observed during evaluation. Understanding these failure modes is essential for contextualizing quantitative results and identifying directions for future improvement. We identify three recurring failure patterns that persist despite the overall strong performance of our unified architecture.

#### 4.3.1 Failure mode 1: visually similar object confusion

The first failure mode involves confusion between visually similar objects particularly when they share similar shapes, colors or contextual placements. For example, in an image containing both a “bowl” and a “cup” placed adjacent to each other on a table, our model generated the French translation “un bol de fruits” (a bowl of fruits) when the English caption correctly specified “a cup of coffee.” The visual features extracted from the region corresponding to the cup shared sufficient similarity with bowl representations in the training data that the cross-modal attention mechanism assigned comparable weights to both objects, leading to semantic substitution. This confusion arises from two interrelated causes. First, the spatial resolution of our visual encoder produces 7 × 7 feature maps where adjacent objects may fall within the same grid cell causing feature blending. Second, the MS-COCO training data contains numerous examples of bowls and cups in similar contexts (kitchen scenes, dining tables), which may not provide sufficient discriminative signal to separate these categories in feature space. Similar confusion patterns were observed between “dog” and “cat” in images where only partial views were visible and between “car” and “bus” in distant street scenes.

#### 4.3.2 Failure mode 2: attribute hallucination in low-resource languages

The second failure mode manifests as attribute hallucination when translating into lower-resource languages within our set particularly Arabic and Hindi. In one instance, an image showing “two children playing with a red ball” generated the Hindi translation “एक बच्चा लाल गेंद से खेल रहा है” (one child playing with a red ball), correctly preserving the color attribute but incorrectly altering the count. In another case, an image captioned “a woman holding an umbrella” produced Arabic translation “مظلة يمسك رجل” (a man holding an umbrella), correctly preserving the action and object but incorrectly assigning gender. Analysis of attention patterns reveals that while visual features for color (red) were correctly attended to in both English captioning and translation stages, the count information (two children) and gender information (woman) received weaker visual grounding. The language-token conditioning mechanism successfully adapted to syntactic structures of Hindi and Arabic as evidenced by correct word order and morphological markings but could not compensate for insufficiently grounded visual attributes. This suggests that our cross-modal alignment mechanism preserves object identity more robustly than object attributes and that attribute grounding requires additional supervisory signals during training.

#### 4.3.3 Failure mode 3: spatial relationship misinterpretation

The third failure mode involves misinterpretation of spatial relationships between objects particularly prepositions such as “behind,” “next to,” and “under.” For an image showing “a cat sitting behind a laptop,” our model generated the Spanish translation “un gato sentado junto a una computadora portátil” (a cat sitting next to a laptop), preserving object identities but flattening the three-dimensional spatial relationship into a simpler co-occurrence. The visual features from the 7 × 7 grid place both cat and laptop in adjacent spatial positions, but the network fails to encode the depth ordering that distinguishes “behind” from “next to.” This limitation stems from the inherent difficulty of encoding three-dimensional spatial relationships from two-dimensional image projections compounded by the relatively coarse 7 × 7 spatial grid of our visual encoder. While positional encodings preserve absolute spatial positions within the grid, relative depth relationships require more sophisticated reasoning about occlusion and perspective that our current architecture does not explicitly model. Similar failures occurred with “under” confused with “next to” and “behind” confused with “beside” across multiple language pairs indicating that spatial relationship errors are modality-specific rather than language-specific.

#### 4.3.4 Implications and mitigation strategies

These failure modes reveal important limitations of our current architecture that were not apparent from aggregate metrics alone. The visually similar object confusion suggests the need for higher-resolution visual features or object-centric representations that better preserve discriminative information. The attribute hallucination in low-resource languages indicates that attribute grounding requires more explicit supervision potentially through auxiliary tasks that predict object attributes directly from visual features. The spatial relationship misinterpretation points to fundamental limitations of 2D feature grids for encoding 3D relationships suggesting that future work should explore depth-aware visual encoders or explicit spatial reasoning modules. Importantly, these failures occur in only 4.7% of test examples based on manual inspection of 500 randomly selected translations and cascaded baselines exhibited these errors at 2–3 times higher frequency. The unified architecture's ability to maintain visual grounding throughout the translation pipeline reduces but does not eliminate these failure modes, highlighting directions for continued research in visually grounded multilingual generation.

### 4.4 Ablation-based component contributions

To isolate the contribution of each architectural innovation and validate our design decisions, we conduct a comprehensive ablation study evaluating several variants of our unified model. These ablations systematically remove or modify core components while keeping all other hyperparameters and training conditions identical enabling direct attribution of performance changes to specific architectural elements. Table 1 summarizes the results for both captioning quality measured by CIDEr and translation accuracy measured by average BLEU across all five target languages along with parameter counts to assess computational efficiency.

#### 4.4.1 Contribution of cross-modal alignment

The removal of cross-modal representation alignment leads to the most substantial performance degradation among all ablation variants. Without alignment, CIDEr drops by 8.6 points from 153.2 to 144.6 and average BLEU decreases by 1.6 points from 41.1 to 39.5. This confirms that the alignment mechanism is critical for preserving visual semantics throughout the generation pipeline. Qualitative analysis of the w/o alignment variant reveals increased frequency of attribute errors particularly in gender agreement for Arabic and French count inaccuracies for Hindi and color misattributions across all languages. The parameter difference between the full model and this variant is minimal at 8 million parameters indicating that the performance gain comes from architectural effectiveness rather than additional capacity. The cross-modal alignment mechanism enables direct gradient flow from translation objectives back to visual feature extraction ensuring that visual representations are optimized for the downstream multilingual task rather than for captioning alone. This joint optimization explains why its removal disproportionately affects translation quality across all language pairs with the largest degradation observed in morphologically rich languages Arabic and Hindi where visually grounded attributes are essential for correct inflection.

#### 4.4.2 Contribution of language token conditioning

Removing language token conditioning and replacing it with separate decoders for each target language produces an interesting trade-off. The separate decoders variant achieves marginally higher CIDEr at 152.8 compared to the full model's 153.2, a difference within statistical noise and average BLEU of 40.5 compared to 41.1 actually slightly lower than the full model. However, this variant requires 312 million parameters representing a 43% increase over the full model's 218 million parameters. This demonstrates that language token conditioning achieves comparable or better performance with substantially improved parameter efficiency. The language token conditioning strategy enables knowledge sharing across languages through shared transformer layers and attention mechanisms while language-specific tokens provide the necessary conditioning to adapt to diverse syntactic and morphological requirements. The parameter efficiency gain is particularly important for deployment scenarios requiring support for many languages where separate decoders would scale linearly with language count while our approach maintains constant parameter count regardless of the number of target languages supported.

#### 4.4.3 Contribution of joint training

The variant without joint training where the caption decoder and translation decoder are trained separately in a two-stage process shows significant degradation with CIDEr dropping 2.4 points to 150.8 and average BLEU dropping 1.8 points to 39.3. This confirms our hypothesis that end-to-end optimization provides beneficial regularization across tasks. In the disjoint training regime, the caption decoder optimizes solely for English captioning quality without considering how its representations will be used for translation while the translation decoder must work with fixed caption representations that may not preserve information critical for multilingual generation. Joint training enables the visual encoder and caption decoder to develop representations that balance the competing demands of fluent English generation and preservable visual semantics for translation. The performance gap is most pronounced for attributes like spatial relationships and object counts which are essential for translation but may be under-specified in English captions that assume shared visual context. This finding supports our core thesis that unified architectures outperform cascaded pipelines precisely because they maintain visual grounding throughout the entire generation process.

#### 4.4.4 Comparison with late fusion baseline

The late fusion variant which integrates visual features only at the final layer of the translation decoder rather than through cross-attention in every layer achieves CIDEr of 147.9 and average BLEU of 38.6 substantially below the full model. This confirms that shallow integration of visual information is insufficient for preserving fine-grained visual semantics. The late fusion approach common in earlier multimodal translation systems allows visual features to influence only the final token predictions without propagating visual grounding through the deeper layers where linguistic structure is constructed. The performance gap between late fusion and our full model demonstrates that visual grounding must be integrated throughout the decoding process to influence syntactic decisions such as agreement, word order and morphological inflection. Languages with freer word order like Hindi particularly benefit from per-layer visual integration as evidenced by the larger performance gap for Hindi (3.2 BLEU points) compared to more rigid languages like French (1.8 BLEU points).

#### 4.4.5 Summary of ablation findings

The ablation study confirms that each component of our unified architecture contributes meaningfully to overall performance. Cross-modal alignment provides the largest individual contribution particularly for attribute preservation and morphologically rich languages. Language token conditioning enables efficient multilingual scaling without sacrificing quality reducing parameter requirements by 43% compared to separate decoders. Joint training ensures that visual representations are optimized for the complete caption-to-translation pipeline rather than for captioning alone. These findings validate our architectural decisions and provide guidance for future work on unified multimodal translation systems.

### 4.5 Linking results to design choices

To provide a deeper understanding of why our model achieves its observed performance, this section explicitly connects experimental results to the architectural design choices demonstrating that our outcomes follow directly from deliberate methodological innovations.

#### 4.5.1 Unified architecture and error propagation mitigation

The fundamental design choice of a unified end-to-end architecture directly explains the 58% reduction in overall error rate compared to cascaded systems. By enabling direct gradient flow from translation objectives back to visual feature extraction, the model learns visual representations optimized for both tasks simultaneously. This design manifests most clearly in the model's ability to resolve captioning ambiguities using visual evidence during translation explaining the 2.2 BLEU point improvement over cascaded baselines.

#### 4.5.2 Cross-modal alignment and attribute preservation

The cross-modal representation alignment mechanism directly accounts for the 39.2% reduction in attribute errors. By fusing visual features with linguistic embeddings through multi-head cross-attention at every decoder layer, the model ensures that visually grounded attributes remain accessible throughout generation. The per-language variation in improvement with Arabic and Hindi showing largest gains validates that alignment specifically benefits morphologically complex languages where attributes influence word formation. The residual connections prove essential as removing them increases attribute errors by 47% in ablation studies.

#### 4.5.3 Language token conditioning and parameter efficiency

The language token conditioning strategy directly explains the 43% parameter reduction while maintaining competitive quality. By prepending learnable language tokens rather than instantiating separate decoders, the model shares all transformer parameters across languages. Analysis confirms tokens have learned to specialize: French tokens attend to gender agreement positions, Arabic tokens focus on verb morphology and Hindi tokens emphasize count-relevant features. The 512-dimensional token design enables direct attention participation without projection contributing to efficiency.

#### 4.5.4 Visual encoder choice and efficiency-performance trade-off

ResNet-50 selection justified in Section 3.2, directly explains observed computational efficiency. Ablation confirms vision transformers achieve only marginal gains ( ≤ 0.2BLEU) at substantial cost (73% longer training, 55% more memory). The 7 × 7 spatial grid producing 49 tokens balances efficiency and resolution: object recognition errors are rare (23% of errors) while spatial relationship errors (12%) reflect anticipated resolution limitations.

#### 4.5.5 Tri-attention ordering and hierarchical grounding

The specific attention ordering, self-attention first, then caption cross-attention, then visual cross-attention, establishes linguistic context before visual refinement. This explains why attribute errors are reduced but not eliminated. The model first generates linguistically derived representations and then attempts visual correction. The 31% residual attribute error rate reflects this deliberate prioritization of fluency over strict visual fidelity.

#### 4.5.6 Hyperparameter choices and convergence behavior

Hyperparameter selections explain stable convergence observed in training curves. The learning rate of 1 × 10^−4^with 4,000-step warmup prevents early instability. Dropout rates of 0.2 (attention) and 0.1 (FFN/embedding) prevent overfitting while maintaining capacity. COMET-based early stopping with patience 10 reflects the design priority of semantic fidelity over n-gram matching.

## 5 Conclusion and future work

This research has presented a unified neural architecture that addresses the persistent challenge of error propagation in MMT through three core technical contributions: an end-to-end trainable framework integrating visual encoding with multilingual generation, a cross-modal representation alignment mechanism for preserving visual semantics and a language-token conditioning strategy enabling efficient multilingual scaling. The experimental results demonstrate that our approach achieves competitive performance in both image captioning and multilingual translation tasks while maintaining computational efficiency.

The empirical validation of our visual encoder choice through comparative analysis with Vision Transformer (*ViT*−*B*/16) and Swin-Tiny architectures confirms that ResNet-50 provides an optimal balance of performance, efficiency and architectural compatibility for our unified framework. The marginal gains from transformer-based encoders ( ≤ 0.2 BLEU, ≤ 0.9 CIDEr) are outweighed by their significant computational overhead (64%–73% longer training time, 42%–55% higher memory consumption) justifying our design decision while acknowledging that future encoder innovations may eventually tip this balance.

This work demonstrates substantial interdisciplinary benefits by bridging computer vision, natural language processing and translation studies. The technical framework provides a foundation for collaborative research that addresses real-world challenges in multimodal communication. For accessibility applications, the system offers potential improvements in generating descriptive captions across multiple languages benefiting visually impaired users in diverse linguistic contexts. In educational technology, the approach could enhance multilingual learning materials by maintaining visual-semantic consistency across language versions. For cultural heritage preservation, the method supports the creation of multilingual descriptions of visual artifacts while preserving contextual meaning. The computational linguistics field benefits from new insights into how visual context influences linguistic choices across different languages. The integration methodology also contributes to human-computer interaction research by demonstrating how complex multimodal systems can be designed for practical deployment (Zhang et al., 2023). The efficiency gains from parameter sharing and conditional decoding provide architectural patterns that could be adapted for other multimodal tasks beyond translation.

While our unified framework achieves universal cross-modal representation learning across multiple languages, multilingual multimodal learning inherently faces uneven data distribution and resource discrepancies between high-resource and low-resource languages. Our current general training strategy prioritizes stable cross-modal neural integration and language-agnostic feature alignment without adopting language-specific adaptive constraints. To further promote balanced cross-lingual representation learning, future work will integrate language-aware weighted optimization, cross-lingual knowledge transfer, and targeted multimodal data enhancement for low-resource languages, so as to improve the model's overall cross-lingual robustness and generalization ability.

Building on this work, several research directions appear promising. Architecturally, exploring hybrid visual encoders that combine the spatial precision of CNNs with the transfer capabilities of vision transformers could enhance both performance and generalization. The recent emergence of efficient ViT variants (e.g., DeiT, EfficientViT) that achieve competitive accuracy with lower computational cost warrants investigation as potential replacements for ResNet-50 in future iterations of our unified architecture. Additionally, integrating more sophisticated VLP objectives during the initial training phase could further improve cross-modal alignment quality without increasing inference complexity.

For language coverage, investigating grapheme-level processing and active learning strategies could expand support for low-resource languages. The current language-token conditioning approach could be extended to support zero-shot translation to unseen languages by learning compositional language representations or leveraging multilingual pre-trained models like mT5 or XLM-R as initialization.

From an evaluation perspective, developing culture-sensitive metrics that assess both linguistic accuracy and contextual appropriateness would better reflect real-world use cases. The integration of human-in-the-loop verification mechanisms could address the current limitations in handling ambiguous or culturally specific content. The principles demonstrated in this work could be extended to other multimodal tasks such as video description, visual question answering and multimodal dialogue systems. The technical approach of joint optimization with conditional decoding provides a framework that could be adapted to various scenarios requiring coordination between visual understanding and linguistic generation.

In summary, this research has established that unified architectures for multimodal translation can achieve competitive performance while addressing key limitations of conventional cascaded systems. The technical contributions provide a foundation for future work in visually grounded multilingual generation with applications spanning translation services, accessibility technology, and cross-cultural communication systems. The empirical validation of architectural choices including the visual encoder comparison ensures that our design decisions are grounded in rigorous analysis and positioned for evolution as the field continues to advance.

Beyond technical advancements in multimodal image-to-text generation, this work contributes to computational neuroscience research by offering an interpretable neural framework for biologically inspired cross-modal integration and multilingual cognitive representation learning. The validated hierarchical cross-modal alignment and token-based neural gating mechanisms provide computational insights into how artificial neural systems achieve human-like multisensory grounded language generation, supporting ongoing journal-focused research on neural computation, multimodal neural circuit modeling, and cognitive language processing.

## Data Availability

The original contributions presented in the study are included in the article/supplementary material, further inquiries can be directed to the corresponding author/s.
